# Intestinal cancer progression by mutant p53 through the acquisition of invasiveness associated with complex glandular formation

**DOI:** 10.1038/onc.2017.194

**Published:** 2017-06-19

**Authors:** M Nakayama, E Sakai, K Echizen, Y Yamada, H Oshima, T-S Han, R Ohki, S Fujii, A Ochiai, S Robine, D C Voon, T Tanaka, M M Taketo, M Oshima

**Affiliations:** 1 Division of Genetics, Cancer Research Institute, Kanazawa University, Kanazawa, Japan; 2 AMED-CREST, AMED, Japan Agency for Medical Research and Development, Tokyo, Japan; 3 Institute of Science and Engineering, Kanazawa University, Kanazawa, Japan; 4 Division of Rare Cancer Research, National Cancer Center Research Institute, Tokyo, Japan; 5 Division of Pathology, Exploratory Oncology Research and Clinical Trial Center, National Cancer Center, Kashiwa Chiba, Japan; 6 Exploratory Oncology Research and Clinical Trial Center, National Cancer Center, Kashiwa Chiba, Japan; 7 Equipe de Morphogenése et Signalisation Cellulaires, Institut Curie, Paris, France; 8 Cancer Research Core, Institute for Frontier Science Initiative, Kanazawa University, Kanazawa, Japan; 9 Department of Clinical Cell Biology and Medicine, Chiba University Graduate School of Medicine, Chiba, Japan; 10 Department of Pharmacology, Kyoto University Graduate School of Medicine, Kyoto, Japan; 11 Division of Experimental Therapeutics, Cancer Research Institute, Kanazawa University, Kanazawa, Japan

## Abstract

Tumor suppressor *TP53* is frequently mutated in colorectal cancer (CRC), and most mutations are missense type. Although gain-of-functions by mutant p53 have been demonstrated experimentally, the precise mechanism for malignant progression in *in vivo* tumors remains unsolved. We generated *Apc*^*Δ716*^ *Trp53*^*LSL•R270H*^ *villin-CreER* compound mice, in which mutant p53^R270H^ was expressed in the intestinal epithelia upon tamoxifen treatment, and examined the intestinal tumor phenotypes and tumor-derived organoids. Mutant *Trp53*^*R270H*^, but not *Trp53*-null mutation accelerated submucosal invasion with generation of desmoplastic microenvironment. The nuclear accumulation of p53 was evident in *Apc*^*Δ716*^ *Trp53*^*R270H/R270H*^ homozygous tumors like human CRC. Although p53 was distributed to the cytoplasm in *Apc*^*Δ716*^ *Trp53*^*+/R270H*^ heterozygous tumors, it accumulated in the nuclei at the invasion front, suggesting a regulation mechanism for p53 localization by the microenvironment. Importantly, mutant p53 induced drastic morphological changes in the tumor organoids to complex glandular structures, which was associated with the acquisition of invasiveness. Consistently, the branching scores of human CRC that carry *TP53* mutations at codon 273 significantly increased in comparison with those of *TP53* wild-type tumors. Moreover, allografted *Apc*^*Δ716*^ *Trp53*^*R270H/R270H*^ organoid tumors showed a malignant histology with an increased number of myofibroblasts in the stroma. These results indicate that nuclear-accumulated mutant p53^R270H^ induces malignant progression of intestinal tumors through complex tumor gland formation and acquisition of invasiveness. Furthermore, RNA sequencing analyses revealed global gene upregulation by mutant p53^R270H^, which was associated with the activation of inflammatory and innate immune pathways. Accordingly, it is possible that mutant p53^R270H^ induces CRC progression, not only by a cell intrinsic mechanism, but also by the generation or activation of the microenvironment, which may synergistically contribute to the acceleration of submucosal invasion. Therefore, the present study indicates that nuclear-accumulated mutant p53^R270H^ is a potential therapeutic target for the treatment of advanced CRCs.

## Introduction

Molecular genetic studies have revealed that genetic alterations in driver genes induce the development of colorectal cancer (CRC) through an adenoma carcinoma sequence.^1, 2, 3^ It has recently been demonstrated that cumulative mutations in *APC*, *KRAS*, *SMAD4* and *TP53* in human intestinal cell-derived organoids are associated with the development of transplanted tumors in immunodeficient mice.^4, 5^ However, despite these findings, the precise functional role of each driver gene mutation in the malignant progression is still not fully understood. Moreover, precisely how the respective genetic alterations in tumor cells contribute to the generation of the microenvironment, which is an important factor in cancer progression, remains to be elucidated. In this regard, mouse genetic studies remain a powerful complementary approach that enables detailed *in vivo* experimentation and observations for delineating the mechanistic basis of tumorigenesis.

The *TP53* is one of the most frequently mutated genes in cancer.^6, 7^ The target of p53 regulates many processes that prevent tumorigenesis, including cell cycle arrest, DNA repair and senescence.^8^ Notably, 74% of p53 mutations are missense mutations, which results in the formation of a mutant protein.^9, 10^ Mouse genetic studies have indicated that missense mutations in *Trp53* at codon 172 and 270 cause adenocarcinomas in the lung and intestine, which are not developed in *Trp53*-null mutant mice, indicating that mutant p53 induces tumors in epithelial organs by a gain-of-function mechanism.^11, 12^ Furthermore, it has been shown that mutant p53 contributes to the invasion and metastasis of rhabdomyosarcoma,^13^ pancreatic cancer^14^ and sporadic and chemically induced colitis-associated intestinal tumors.^15, 16^ Mechanistically, mutant p53 promotes tumorigenesis by the activation of hepatocyte growth factor and platelet-derived growth factor receptor β signaling.^17, 18^ Moreover, mutant p53 alters gene expression at a global level through epigenetic mechanisms, namely by recruiting the SWI/SNF complex to remodel the chromatin of gene promoters,^19^ and through the induction of MLL1, MLL2 and MOZ to modify histone methylation and acetylation.^20^ However, the contribution of mutant p53 to tumor invasion and metastasis during *in vivo* tumorigenesis and the underlying mechanisms remains unclear.

In the present study, we generated *Apc*^*Δ716*^ and *Trp53*^*R270H*^ compound mutant mice to examine the effect of mutant p53^R270H^ on *Apc* mutation-induced intestinal tumors. *Apc*^*Δ716*^ mice develop benign intestinal tumors through β-catenin stabilization and subsequent Wnt signaling activation.^21^ Notably, mutant p53 strongly accumulates in the nuclei of tumor cells at the invasion front where desmoplastic microenvironment is generated. We observed that mutant p53 induces drastic morphological changes in tumor organoids with the acquisition of invasiveness, which is related to the increased branching of *in vivo* mouse tumors and human CRC. Mutant p53 also induced a marked shift in the transcriptome, which caused a significant activation of inflammatory and innate immune pathways together with Wnt/β-catenin signaling. Therefore, the present study indicates that mutant p53^R270H^ induces intestinal tumor invasion through the acquisition of invasiveness with a complex tumor gland architecture, and mutant p53-induced activation of the inflammatory pathways and Wnt signaling may cooperatively contribute to these malignant phenotypes.

## Results

### Induction of submucosal invasion of intestinal tumors by *Trp53* mutation

We generated *Apc*^*Δ716*^ *Trp53*^*+/LSL•R270H*^ *villin-CreER* and *Apc*^*Δ716*^ *Trp53*^*LSL•R270H/LSL•R270H*^ *villin-CreER* compound mice (hereafter, *Apc*^*Δ716*^ *Trp53*^*+/flox*^ and *Apc*^*Δ716*^ *Trp53*^*flox/flox*^). The *Trp53*^*flox*^ allele is functionally *Trp53*-null, and treatment of *Apc*^*Δ716*^ *Trp53*^*+/flox*^ and *Apc*^*Δ716*^ *Trp53*^*flox/flox*^ mice with tamoxifen results in the expression of mutant p53^R270H^ in intestinal tumor cells (hereafter, *Apc*^*Δ716*^ *Trp53*^+/R270H^ and *Apc*^*Δ716*^ *Trp53*^*R270H/R270H*^).

**Figure 1:**
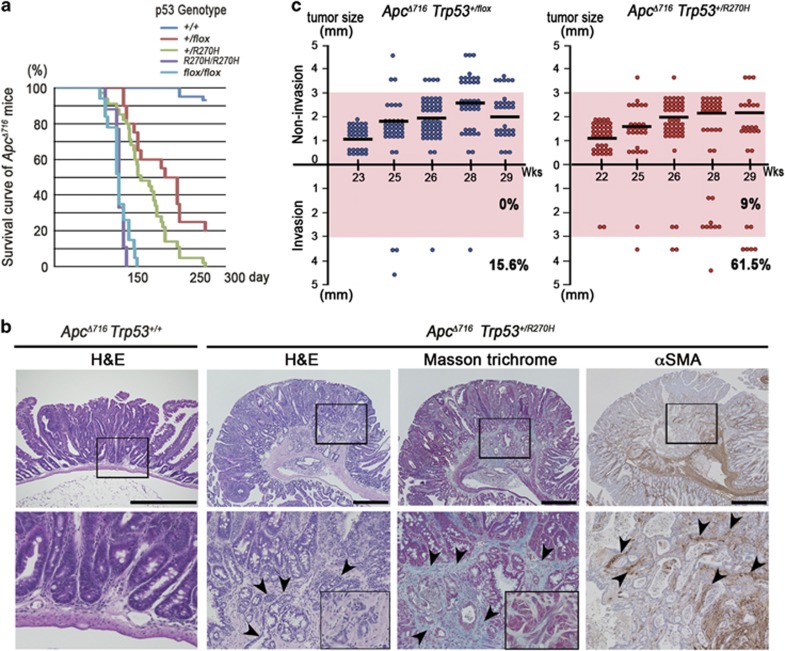
Submucosal invasion of *Apc*^*Δ716*^ intestinal polyps by mutant p53^R270H^. (**a**) The survival curves of each type of *Apc*^*Δ716*^ *Trp53* compound mice are shown. (**b**) Representative photographs *Apc*^*Δ716*^ *Trp53*^*+/+*^ mouse intestinal adenomas (left, H&E) and *Apc*^*Δ716*^ *Trp53*^*+/R270H*^ mouse invasive adenocarcinomas (right, H&E, Masson trichrome and αSMA immunostaining). The bottom photographs are enlarged images of the top (boxed area). The inset in the Masson trichrome staining image (bottom) shows a magnified view of dysplastic tumor cells. The arrowheads indicate invading tumor cells (H&E), collagen fibers (Masson Trichrome) and myofibroblasts (αSMA). Bars, 500 μm. (**c**) Size classification of intestinal tumors of *Apc*^*Δ716*^ *Trp53*^*+/flox*^ mice (left) and *Apc*^*Δ716*^ *Trp53*^*+/R270H*^mice (right) at indicated weeks of ages. The numbers of invasive and non-invasive tumors were separately scored. Each dot indicates an individual tumor. Horizontal bars indicate the mean polyp sizes. The percentages of invasive tumors are indicated for tumors <3 mm (pink area) and ⩾3 mm in diameter (outside of pink area). Wks, weeks.

The mean lifespan of *Apc*^*Δ716*^ *Trp53*^*R270H/R270H*^ mice was comparable to that of *Apc*^*Δ716*^ *Trp53*^*flox/flox*^ mice, as they developed lymphomas owing to the loss of wild-type *Trp53* (Figure 1a). *Apc*^*Δ716*^ *Trp53*^*+/R270H*^ heterozygous mice survived longer than *Apc*^*Δ716*^ *Trp53*^*R270H/R270H*^ mice, which allowed them to develop intestinal adenocarcinomas with submucosal invasion with desmoplasia consisting of collagen fibers and α-smooth muscle actin (αSMA)-positive myofibroblasts (Figure 1b, arrowheads). The mean polyp size in the *Apc*^*Δ716*^ *Trp53*^*+/flox*^ and *Apc*^*Δ716*^ *Trp53*^*+/R270H*^ mice (five mice from 22 to 29 weeks of ages for each genotype) was 1.97±0.82 mm and 1.95±0.74 mm, respectively, with no significant difference. However, the incidence of invasive tumors was markedly increased in *Apc*^*Δ716*^ *Trp53*^*+/R270H*^ mice compared with *Apc*^*Δ716*^ *Trp53*^*+/flox*^ mice (Figure 1c), which coincided with the shorter lifespan (161 days vs 202 days in *Apc*^*Δ716*^ *Trp53*^*+/R270H*^ and *Apc*^*Δ716*^ *Trp53*^*+/flox*^, respectively, by the log-rank test, *P*=0.0162). Immunohistochemical analysis revealed that the expression of mesothelin, an invading CRC cell marker,^22^ and snail2, an epithelial-mesenchymal transition marker,^23, 24^ was induced at the submucosal invasive area of *Apc*^*Δ716*^ *Trp53*^*+/R270H*^ adenocarcinomas, but it was not detected in the invasive area of *Apc*^*Δ716*^ *Trp53*^*+/flox*^ tumors (Supplementary Figure 1a). In addition, the expression of the stem cell marker *Cd44* was significantly increased in the tumor cells of invasive areas (Supplementary Figure 1b). Accordingly, it is possible that the mutant p53^R270H^ causes intestinal tumor invasion through the induction of invasiveness and the undifferentiated characteristics of the tumor cells by a gain-of-function mechanism. In contrast, there was essentially no difference in the Ki67 labeling indices among *Apc*^*Δ716*^ *Trp53*^*+/+*^, *Apc*^*Δ716*^ *Trp53*^*+/R270H*^ and *Apc*^*Δ716*^ *Trp53*^*R270H/R270H*^ tumors. Likewise, apoptotic cells were rarely found in the tumors of either genotype (Supplementary Figure 1c and d).

### Nuclear accumulation of p53 in the tumor cells of invasive area

**Figure 2:**
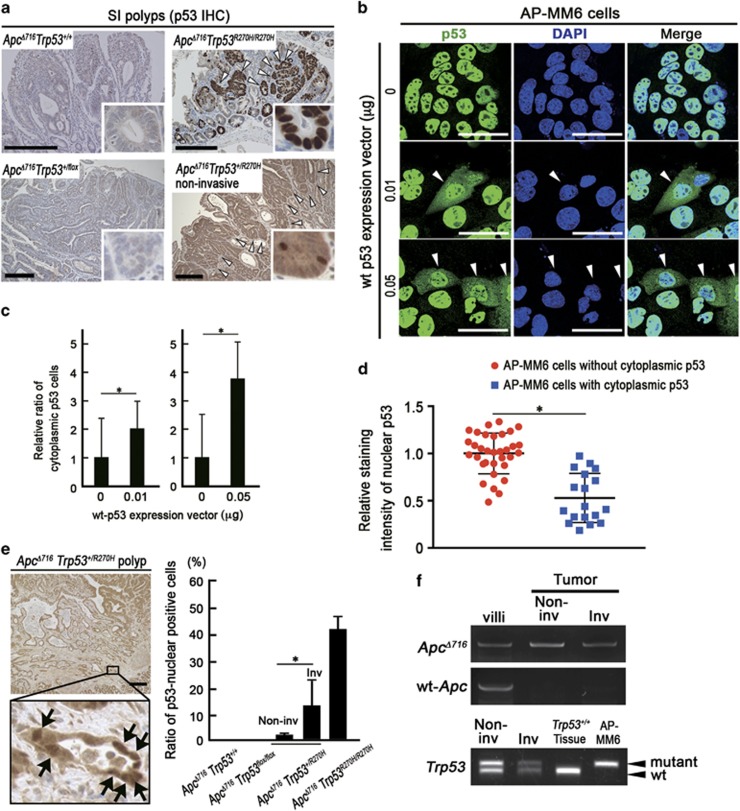
Nuclear localization of p53 in the invasive intestinal tumor cells. (**a**) Representative photographs of immunohistochemistry for p53 in small intestinal (SI) polyps of the indicated genotype mice. The insets show enlarged images. The arrowheads indicate p53 positive tumor cells. Bars, 200 μm. (**b**) Representative photographs of fluorescence immunocytochemistry for p53 (green), nuclear counterstaining with DAPI (blue) and merged images (right) of AP-MM6 cells transfected with the wild-type (wt) p53 expression vector (middle and bottom) and control (top). The arrowheads indicate AP-MM6 cells showing both nuclear and cytoplasmic p53 localization. Bars, 50 μm. (**c**) The ratio of AP-MM6 cells with cytoplasmic p53 distribution after transfection of 0.01 μg (left) or 0.05 μg (right) of wild-type p53 expression vector relative to the control level (mean±s.d.). Asterisks, *P*<0.05. (**d**) Relative staining intensity of nuclear p53 in AP-MM6 cells with or without cytoplasmic distribution of p53 after wt p53 vector transfection (blue and red, respectively). Each dot indicates a single cell. Asterisk, *P*<0.05. (**e**) Representative photographs of immunohistochemistry for p53 in the invasive region of *Apc*^*Δ716*^ *Trp53*^*+/R270H*^ mouse intestinal tumors with low-power magnification (left top) and an enlarged image (left bottom). The arrows indicate tumor cells with nuclear-accumulated p53. Bars, 200 μm. The relative ratio of tumor cells with nuclear-accumulated p53 in the indicated genotypes is shown (mean±s.d.) (right). Asterisk, *P*<0.05. (**f**) An LOH analysis for *Apc* and *Trp53* by LMD-based genomic PCR in the non-invasive (Non-inv) and invasive (Inv) areas of *Apc*^*Δ716*^ *Trp53*^*+/R270H*^ intestinal tumors and normal villi as the controls. The mutant *Trp53* and wild-type *Trp53*-specific bands are indicated as ‘mutant’ and ‘wt’, respectively. Genomic DNA of *Trp53* wild-type mouse tissue and AP-MM6 were used for positive control of wt and mutant *Trp53*, respectively. Note that wild-type (wt) *Apc* was lost in both non-invasive and invasive tumor cells, whereas wild-type *Trp53* is remained in these cells.

A striking nuclear accumulation of p53 was observed in the *Apc*^*Δ716*^ *Trp53*^*R270H/R270H*^ mouse tumors like human CRC with *TP53* mutations, whereas p53 was not detected in the *Apc*^*Δ716*^ *Trp53*^*+/+*^ and *Apc*^*Δ716*^ *Trp53*^*+/flox*^ mouse tumors (Figure 2a and Supplementary Figure 2). Notably, p53 was stabilized and distributed to cytoplasm in *Apc*^*Δ716*^ *Trp53*^*+/R270H*^ heterozygous mouse tumors, suggesting that wild-type p53 suppresses the nuclear accumulation of mutant p53, possibly through a dominant-negative mechanism. To assess this possibility, we established a cell line AP-MM6 from *Apc*^*Δ716*^ *Trp53*^*R270H/R270H*^ mouse intestinal tumors. As anticipated, stabilized mutant p53 was accumulated in the nuclei of most AP-MM6 cells (Figure 2b). Of note, the expression of exogenous wild-type *Trp53* in AP-MM6 cells caused a vector DNA amount-dependent increase of cells with cytoplasmic p53 distribution (Figures 2b and c). Moreover, in the AP-MM6 cells with cytoplasmic p53, the intensity of nuclear p53 significantly decreased (Figure 2d). These results support the idea that wild-type p53 suppresses the nuclear accumulation of mutant p53, although further studies are needed for validation.

Interestingly, however, we found the nuclear accumulation of p53 in the invasion front of *Apc*^*Δ716*^ *Trp53*^*+/R270H*^ mouse tumors where the number of stromal cells increased (Figure 2e). Moreover, wild-type *Trp53* alleles were detected in these cells by laser microdissection-coupled genomic polymerase chain reaction (PCR), excluding the possibility of the lost of wild-type *Trp53* by the loss of heterozygosity in the invasive area (Figure 2f). In contrast, wild-type *Apc* is lost in *Apc*^*Δ716*^ tumor cells, which is consistent with the findings from a previous report.^21^ Accordingly, it is possible that the subcellular distribution of mutant p53 in tumor cells is affected by the microenvironment of the invasive area.

### Induction of the complex glandular structure of tumors by mutant p53^R270H^

**Figure 3:**
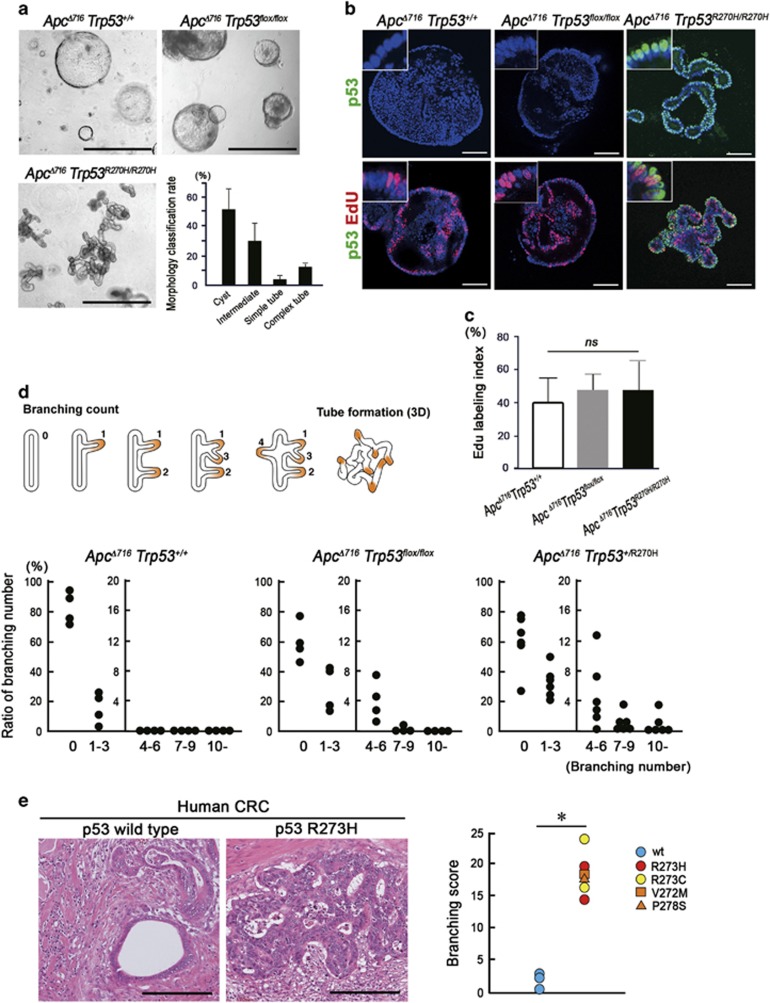
Induction of morphological changes of the tumors by mutant p53^R270H^. (**a**) Representative photographs of the indicated genotype organoids. Bars, 1 mm. The ratios of morphological classifications of *Apc*^*Δ716*^ *Trp53*^*R270H/R270H*^ organoids are shown as a bar graph (mean±s.d.) (bottom right). (**b**) Representative confocal microscopy images of *Apc*^*Δ716*^ *Trp53*^*+/+*^ (left), *Apc*^*Δ716*^ *Trp53*^*flox/flox*^ (center) and *Apc*^*Δ716*^ *Trp53*^*R270H/R270H*^ tumor organoids (right). Fluorescence immunostaining for p53 (green, top) and double immunostaining for p53 and EdU (green and red, respectively, bottom) with DAPI staining (blue). The insets show enlarged images. Bars, 100 μm. (**c**) The EdU labeling index of tumor organoids is shown (mean±s.d.). *ns*, not significant. (**d**) A schematic diagram of the representative patterns of intestinal tumor branching leading to tube formation (top). The ratio of the branching number classification of *Apc*^*Δ716*^ *Trp53*^*+/+*^, *Apc*^*Δ716*^ *Trp53*^*flox/flox*^ and *Apc*^*Δ716*^ *Trp53*^*+/R270H*^ intestinal tumors are shown (bottom). Each dot represents the average rate (%) of the branching number in the individual mouse tumors. (**e**) Representative micrographs of human CRC with *TP53* wild-type and R273H mutation (left, H&E). *Bars*, 200μm. Branching scores of human CRC with *TP53* mutations around codon 273 (red, yellow and orange) compared with *TP53* wild-type cases (blue) are shown (right). Asterisk, *P*<0.05.

To investigate the role of nuclear-accumulated p53^R270H^ in submucosal invasion, we examined intestinal tumor-derived organoids. In this study, we analyzed *Apc*^*Δ716*^ *Trp53*^*R270H/R270H*^ tumor organoids because immunocytochemical staining showed a predominant p53 localization in the nucleus, whereas the nuclear accumulation was limited and cytoplasmic stabilization of p53 was not detected in the *Apc*^*Δ716*^ *Trp53*^*+/R270H*^ organoids (Supplementary Figure 3a). Morphologically, the organoids derived from *Apc*^*Δ716*^ *Trp53*^*+/+*^ and *Apc*^*Δ716*^ *Trp53*^*flox/flox*^ tumors showed round cystic structures, reflecting the uniform undifferentiated state caused by Wnt signaling activation in all cells (Figure 3a and Supplementary Figure 3b).^25^ *Apc*^*Δ716*^ *Kras*^*G12D*^ and *Apc*^*Δ716*^ *Tgfbr2*^*ΔIEC*^ tumor organoids also showed similar cystic morphologies. In contrast, *Apc*^*Δ716*^ *Trp53*^*R270H/R270H*^ tumor organoids frequently formed simple as well as complex tube structures from Day 2 after each passage (Figure 3a and Supplementary Figure 4a), indicating that *Trp53*^*R270H*^ mutation induces morphological changes by a gain-of-function. EdU (5-ethynyl-2’-deoxyuridine) labeling experiments showed no significant difference in the proliferation rate among *Apc*^*Δ716*^ *Trp53*^*+/+*^, *Apc*^*Δ716*^ *Trp53*^*flox/flox*^ and *Apc*^*Δ716*^ *Trp53*^*R270H/R270H*^ tumor organoids (Figures 3b and c).

To address the relationship between the complex glandular architecture of *Apc*^*Δ716*^ *Trp53*^*R270H/R270H*^ organoids and the distinct histology of the *in vivo* tumors, we scored the branching of the intestinal tumor glands of each genotype mouse. Notably, the branching rate in *Apc*^*Δ716*^ *Trp53*^*+/R270H*^ mouse tumors was markedly higher than that in *Apc*^*Δ716*^ *Trp53*^*+/+*^ or *Apc*^*Δ716*^ *Trp53*^*flox/flox*^ counterparts (Figure 3d). Although the tumor size was small in *Apc*^*Δ716*^ *Trp53*^*R270H/R270H*^ mice because of their short lifespan (Figure 1a), we confirmed that the branching rate tended to be higher in the *Apc*^*Δ716*^ *Trp53*^*R270H/R270H*^ mouse tumors (polyp diameter 1–2 mm) than in size-matched *Apc*^*Δ716*^ *Trp53*^*flox/flox*^ tumors (Supplementary Figure 3c). We next examined the branching frequency in human CRC carrying *TP53* mutations around codon 273 (corresponding to codon 270 in mice). Importantly, CRC with *TP53* mutations showed a significantly increased branching score compared with that in *TP53* wild-type (Figure 3e). These results suggest that mutant p53 can promote structural atypia of intestinal tumors through the construction of a complex glandular structure.

### Acquisition of invasiveness of tumor glands expressing p53^R270H^

**Figure 4:**
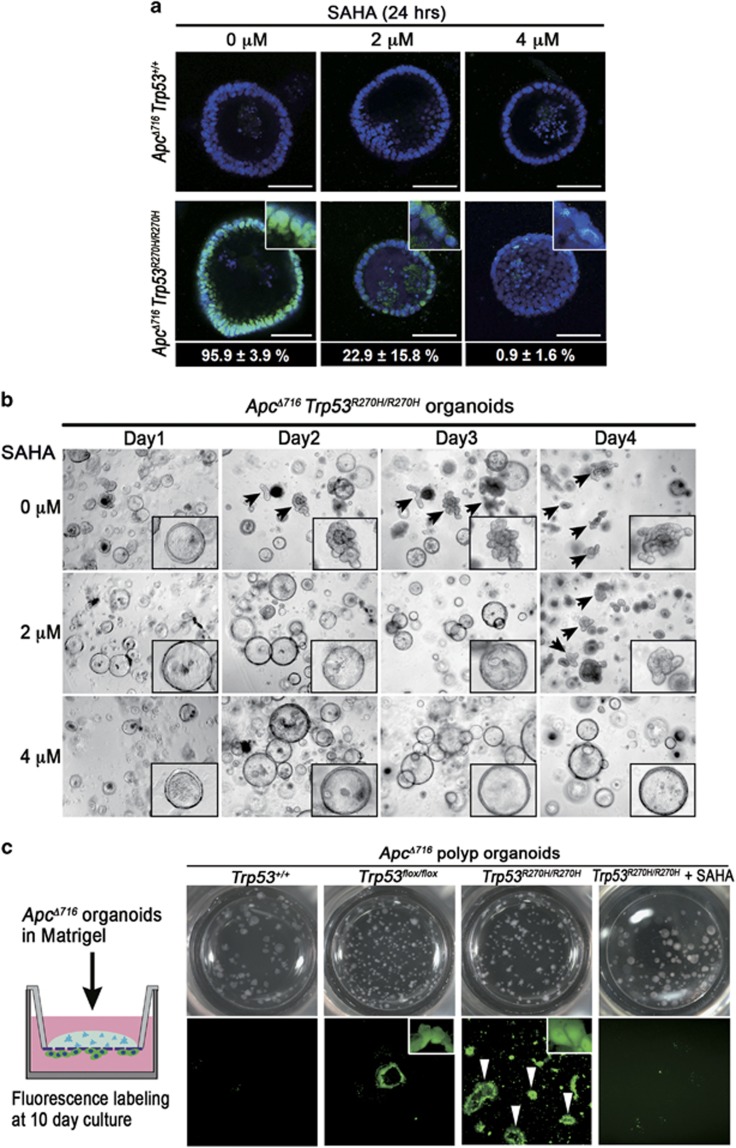
Complex tube structure formation and the acquisition of invasiveness by mutant p53^R270H^. (**a**) Representative confocal microscopy images of fluorescence immunostaining for p53 (green) with nuclear counterstaining DAPI (blue) of *Apc*^*Δ716*^ *Trp53*^*+/+*^ (top) and *Apc*^*Δ716*^ *Trp53*^*R270H/R270H*^ organoids (bottom) treated with the indicated concentration of SAHA. Insets indicate enlarged images. Bars, 100 μm. Ratios of the cells with nuclear-accumulated p53 in the *Apc*^*Δ716*^ *Trp53*^*R270H/R270H*^ organoids treated with SAHA at each concentration are indicated in the bottom (mean%±s.d.%). (**b**) Representative dissecting microscopy photographs of *Apc*^*Δ716*^ *Trp53*^*R270H/R270H*^ organoids at Days 1–4 after SAHA treatment with the indicated concentration. The arrows indicate organoids with complex tubular structures. Insets indicate enlarged images. (**c**) An illustration of the organoid invasion assay (left). Representative dissecting microscopy photographs of upper wells (top) and fluorescence microscopy images of the bottom surface of transwells (bottom) of the indicated genotype organoids and SAHA-treated *Apc*^*Δ716*^ *Trp53*^*R270H/R270H*^ organoids. The arrowheads indicate invading tumor cells forming a glandular structure.

It has been reported that mutant p53 proteins are stabilized by interaction with the HSP90 chaperone mechanism, which inhibits MDM2 activity.^26, 27^ As HSP90 is positively regulated by histone deacetylase 6, targeting histone deacetylase 6 using suberoylanilide hydroxamic acid (SAHA) can destabilize the mutant p53 proteins.^28^ We confirmed that SAHA treatment abolished the nuclear accumulation of p53^R270H^ in *Apc*^*Δ716*^ *Trp53*^*R270H/R270H*^ organoids in a dose-dependent manner (Figure 4a). Importantly, SAHA treatment blocked the formation of complex glandular structures and reverted the morphology of *Apc*^*Δ716*^ *Trp53*^*R270H/R270H*^ organoids to the round cystic typical of *Apc*^*Δ716*^ mouse tumor organoids (Figure 4b and Supplementary Figure 4a). These results suggest that p53^R270H^ is required to maintain the complex tubular morphology of the organoids.

We next investigated the invasive capacity of organoids of different *Trp53* genotypes using a transwell invasion assay. The results showed that *Apc*^*Δ716*^ *Trp53*^*+/+*^ organoids have no invasive capacity, whereas *Apc*^*Δ716*^ *Trp53*^*flox/flox*^ organoids displayed only limited invasive properties (Figure 4c). In contrast, significant invasion was observed for the *Apc*^*Δ716*^ *Trp53*^*R270H/R270H*^ organoids that retained their tubular structure during invasion (Figure 4c, arrowheads). Importantly, treatment of *Apc*^*Δ716*^ *Trp53*^*R270H/R270H*^ organoids with SAHA effectively inhibited invasion (Figure 4c and Supplementary Figure 4b). These results indicate that p53^R270H^-induced complex gland formation is accompanied by the acquisition of invasive properties, although the possibility of a p53 degradation-independent mechanism by SAHA remains to be investigated.

### Malignant tumor development by *Apc^Δ716^ Trp53^R270H/R270H^* tumor organoid transplantation

**Figure 5:**
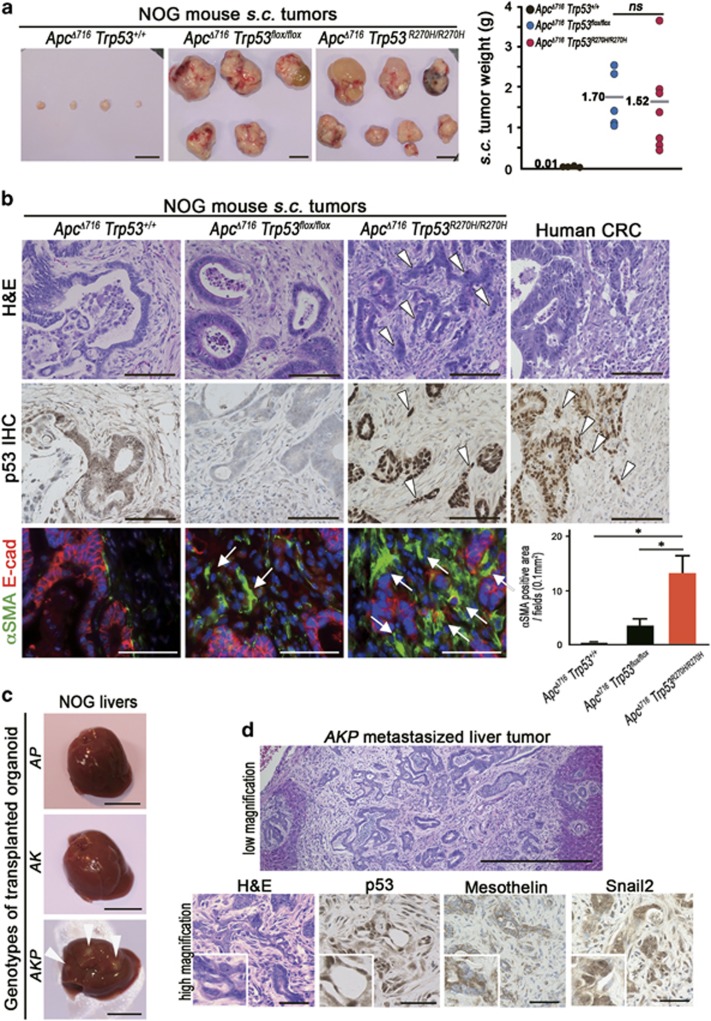
Malignant tumor formation in NOG mice by p53^R270H^ organoids. (**a**) Representative macroscopic photographs of the organoid-derived s.c. tumors in NOG mice at 4 months after transplantation (left, three photographs). Bars, 1 cm. The weights of s.c. tumors for each genotype with the average are plotted in a dot graph (right). ns, not significant. (**b**) Representative histology photographs of organoid-derived s.c. tumors and human CRC (stage IIIB). H&E (top) and immunohistochemistory for p53 (middle) and double fluorescence immunostaining for αSMA (green) and E-cadherin (red) (bottom). The arrowheads indicate the sheets of tumor cells in *Apc*^*Δ716*^ *Trp53*^*R270H/R270H*^ s.c. tumors and human CRC. The arrows indicate αSMA-positive myofibroblasts. Bars, 100 μm. The αSMA-positive areas for each tumor genotype determined using fluorescence immunohistochemistry are shown as a bar graph (mean±s.d.). Asterisks, *P*<0.05. (**c**) The macroscopic photographs of NOG mouse livers at 1 month after injection of the organoids into the spleen. The arrowheads indicate metastasized tumors. *AP*, *Apc*^*Δ716*^ *Trp53*^*R270H/R270H*^; *AK*, *Apc*^*Δ716*^ *Kras*^*G12D*^; *AKP*, *Apc*^*Δ716*^ *Kras*^*G12D*^ *Trp53*^*+/R270H*^. Bars, 1 cm. (**d**) Representative photographs of metastasized *AKP* organoid-derived tumors in NOG mouse livers. H&E staining (top and bottom left) and immunohistochemistry for p53, mesothelin and Snail2 (bottom, left to right). Bars, 500 μm (top) and 50 μm (bottom).

Given that *Apc*^*Δ716*^ *Trp53*^*R270H/R270H*^ mice succumb prior to the development of malignant intestinal adenocarcinoma, we transplanted *Apc*^*Δ716*^ *Trp53*^*R270H/R270H*^ tumor organoids subcutaneously (s.c.) into immunodeficient NOG mice to further examine their tumorigenicity. Control *Apc*^*Δ716*^ *Trp53*^*+/+*^ tumor organoids survived for 4 months after transplantation without forming palpable tumors (Figure 5a). In contrast, both *Apc*^*Δ716*^ *Trp53*^*flox/flox*^ and *Apc*^*Δ716*^ *Trp53*^*R270H/R270H*^ organoids formed large tumors of comparable sizes. Histologically, residual *Apc*^*Δ716*^ *Trp53*^*+/+*^ allografts and *Apc*^*Δ716*^ *Trp53*^*flox/flox*^ organoid tumors showed mostly cystic structures (Figure 5b). In contrast, *Apc*^*Δ716*^ *Trp53*^*R270H/R270H*^ organoid tumors presented a malignant histology with nuclear p53 accumulation, such as extensive branching of the tumor glands or occasional cell clusters in sheets (Figure 5b, arrowheads). The mean ratios of the cell clusters without gland formation in *Apc*^*Δ716*^ *Trp53*^*flox/flox*^ and *Apc*^*Δ716*^ *Trp53*^*R270H/R270H*^ tumors were 3.1% and 68.8%, respectively. Notably, the αSMA-positive area was increased significantly in the *Apc*^*Δ716*^ *Trp53*^*R270H/R270H*^ s.c. tumor stroma (Figure 5b, arrows and bar graph), suggesting that the microenvironment is activated with increased myofibroblasts by the *Trp53* mutation in cancer cells. These morphological characteristics of *Apc*^*Δ716*^ *Trp53*^*R270H/R270H*^ tumors closely mimic those of moderately differentiated human colon cancer (Figure 5b).^29^

Despite their malignant histology, *Apc*^*Δ716*^ *Trp53*^*R270H/R270H*^ organoid s.c. tumors did not metastasize to distant organs. To assess the metastatic potential of *Trp53*^*R270H*^ cancer cells, we injected tumor-derived organoids into NOG mouse spleens to test their liver metastasis. In this experiment, we used organoids derived from *Apc*^*Δ716*^ *Trp53*^*R270H/R270H*^, *Apc*^*Δ716*^ *Kras*^*G12D*^ and *Apc*^*Δ716*^ *Kras*^*G12D*^ *Trp53*^*+/R270H*^ mouse tumors. Among these genotypes, only *Apc*^*Δ716*^ *Kras*^*G12D*^ *Trp53*^*+/R270H*^ triple mutant organoids developed metastasis foci in the liver (Figure 5c). A histological analysis showed the moderately to poorly differentiated features of the metastasized tumors with p53 nuclear accumulation and the expression of mesothelin and Snail2 (Figure 5d). Collectively, these results indicate that the combination of p53 mutation with Kras activation can induce metastasis of intestinal tumors.

### Expression of a wide range of genes induced by nuclear-accumulated p53^R270H^

**Figure 6:**
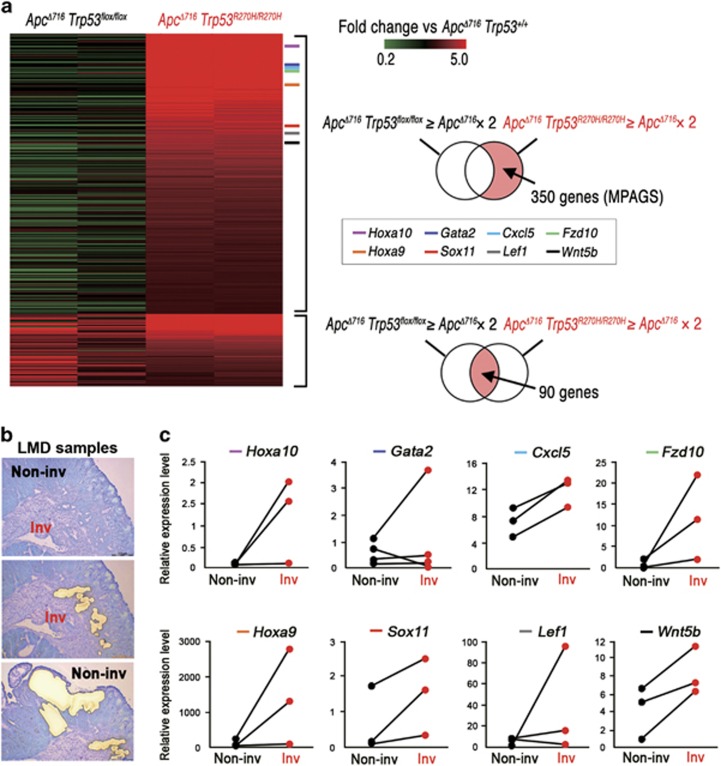
Expression of a wide range of genes by nuclear-accumulated p53^R270H^. (**a**) A heat map of 440 genes that are upregulated in *Apc*^*Δ716*^ *Trp53*^*R270H/R270H*^ organoids more than twofold compared with *Apc*^*Δ716*^ *Trp53*^*+/+*^ organoids. Of 440 genes, 350 were upregulated in *Apc*^*Δ716*^ *Trp53*^*R270H/R270H*^ organoids, but not in *Apc*^*Δ716*^ *Trp53*^*flox/flox*^ organoids, and were designated as the mutant p53-activated gene set (MPAGS), whereas the other 90 genes were upregulated in both *Apc*^*Δ716*^ *Trp53*^*R270H/R270H*^ and *Apc*^*Δ716*^ *Trp53*^*flox/flox*^ organoids. (**b**) Representative photographs of an intestinal tumor after the collection of invasive tumor cells (middle) and non-invasive tumor cells (bottom) by LMD. (**c**) Relative mRNA expression of eight selected genes from the MPAGS in non-invasive area (Non-inv) and invasive area (Inv) of *Apc*^*Δ716*^ *Trp53*^*+/R270H*^ mouse tumors. The lines connect the real-time RT-PCR results of the non-invasion (closed) and invasion (red) areas of the same tumors.

To examine the molecular mechanism through which p53^R270H^ promotes malignant progression, we performed RNA Sequencing of the respective *Trp53* genotype organoids. A clustering analysis showed a gene cluster specifically upregulated in the *Apc*^*Δ716*^ *Trp53*^*R270H/R270H*^ tumor organoids (Supplementary Figure 5a, asterisk). Of the 440 genes elevated more than twofold in the *Apc*^*Δ716*^ *Trp53*^*R270H/R270H*^ organoids relative to *Apc*^*Δ716*^ *Trp53*^*+/+*^, 350 genes were not upregulated in *Apc*^*Δ716*^ *Trp53*^*flox/flox*^ organoids, and were thus designated as ‘a mutant p53-activated gene set (MPAGS)’ (Figure 6a and Supplementary Table 1). We selected eight genes (*Hoxa10*, *Gata2*, *Cxcl5*, *Fzd10*, *Hoxa9*, *Sox11*, *Lef1* and *Wnt5b*) from MPAGS for the validation of a sequencing analysis by real-time reverse transcribed (RT)-PCR and confirmed the upregulation of these genes in *Apc*^*Δ716*^ *Trp53*^*R270H/R270H*^ organoids compared with *Apc*^*Δ716*^ *Trp53*^*+/+*^ and *Apc*^*Δ716*^ *Trp53*^*flox/flox*^ (Supplementary Figure 5b). As the nuclear accumulation of p53 is restricted to the submucosal invasive area of *Apc*^*Δ716*^ *Trp53*^*+/R270H*^ tumors (Figure 2e), we separately collected tumor cells from the invasive and non-invasive areas of *Apc*^*Δ716*^ *Trp53*^*+/R270H*^ mouse tumors by laser microdissection (Figure 6b), and analyzed the expression of the eight validated genes by real-time RT-PCR (Figure 6c). Notably, the expression of these genes was elevated in the invasive areas compared with their non-invasive counterparts, indicating that the nuclear localization of p53^R270H^ causes a transcriptomic shift in tumor cells.

Recent studies show that mutant p53 induces the epigenetic modification of chromatin.^19, 20^ We thus analyzed the accessibilities of the promoter regions of validated MPAGS genes. The data showed that the promoter regions of *Hoxa9*, *Hoxa10*, *Gata2* and *Lef1* genes were substantially more sensitive to the nuclease treatment in *Apc*^*Δ716*^ *Trp53*^*R270H/R270H*^ tumor cells than in *Apc*^*Δ716*^ *Trp53*^*+/+*^ and *Apc*^*Δ716*^ *Trp53*^*flox/flox*^ (Supplementary Figure 5c). These results indicate that mutant p53 induces a more opened chromatin structure, which rendered gene promoters more accessible to transcription factors, at least in a portion of MPAGS.

### Activation of inflammatory and innate immune signaling in *Apc*^*Δ716*^*Trp53*^*R270H/R270H*^ organoids

**Figure 7:**
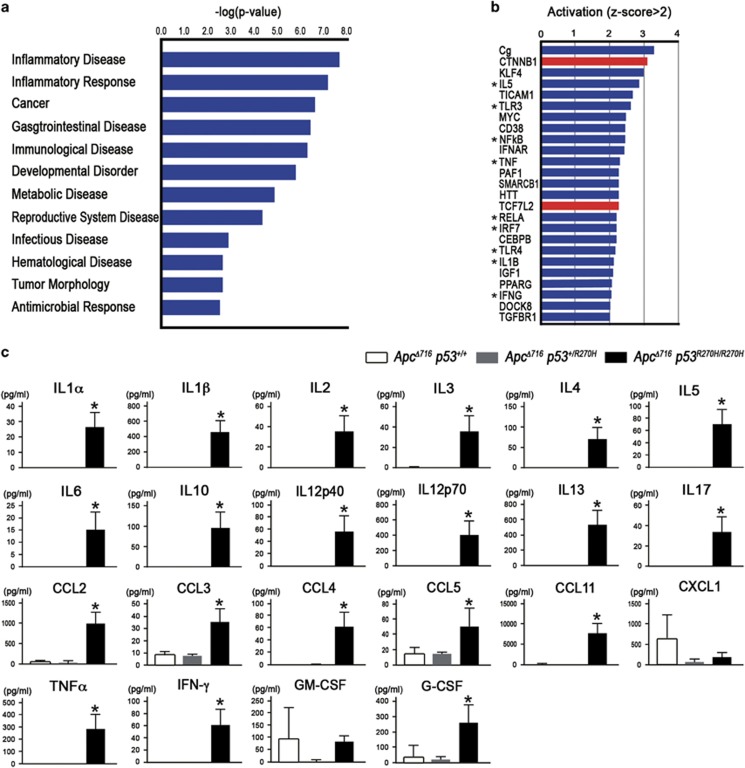
Activation of inflammatory and innate immune pathways in tumor cells by p53^R270H^. (**a**) The results of a gene ontology (GO) analysis using MPAGS showing significantly activated disease category in *Apc*^*Δ716*^ *Trp53*^*R270H/R270H*^ organoids aligned by *P*-values. (**b**) The results of an ingenuity pathway analysis (IPA) showing significantly upregulated pathways in *Apc*^*Δ716*^ *Trp53*^*R270H/R270H*^ organoids aligned by *P*-values. Asterisks, inflammatory and innate immune pathways. *Red bars*, Wnt/β-catenin signaling. (**c**) Medium concentration of cytokines and chemokines in the organoid cultures for the indicated genotypes are shown as bar graphs (mean±s.d.). Asterisks, *P*<0.05.

A gene ontology term analysis for the disease category using MPAGS indicated a significant activation of ‘Inflammatory Disease’ and ‘Inflammatory Response’ in addition to ‘Cancer’ in the *Apc*^*Δ716*^ *Trp53*^*R270H/R270H*^ tumor cells (Figure 7a). Consistently, Ingenuity Pathway Analysis indicated that inflammatory and innate immune signaling pathways were significantly activated in *Apc*^*Δ716*^ *Trp53*^*R270H/R270H*^ tumor organoid cells (Figure 7b, asterisks). We thus examined medium concentrations of cytokines and chemokines in the respective genotype organoid cultures. Consistently, the levels of most cytokines and chemokines were significantly elevated in *Apc*^*Δ716*^ *Trp53*^*R270H/R270H*^ organoid culture media (Figure 7c). It is possible that *Trp53* mutation in cancer cells triggers the generation of a microenvironment through the secretion of inflammatory factors (Supplementary Figure 6).

Moreover, we found that the Wnt/β-catenin signaling pathway was also activated in p53^R270H^ tumor organoids (Figure 7b, red bars). It has been shown that the concurrent activation of NF-κB and Wnt signaling pathways promotes a stem cell-like state.^30^ Accordingly, it is also conceivable that the *Trp53* mutation induces stemness or an undifferentiated status of tumor cells through the simultaneous activation of inflammatory pathways and Wnt signaling (Supplementary Figure 6).

## Discussion

The accumulation of driver gene mutations promotes cancer development and malignant progression.^2^ Recent studies have shown that the transformation of human normal colonic stem cells can be achieved by the accumulation of *APC*, *KRAS*, *SMAD4* and *TP53*-null mutations.^4, 5^ However, these transformed organoids failed to show a fully metastatic malignant behavior of human CRC.^4, 31^ In contrast, we herein show that triple mutations in *Apc*^*Δ716*^, *Kras*^*G12D*^ and *Trp53*^*R270H*^ showed liver metastasis after being injected into the spleen of NOG mice. Accordingly, it is possible that *Trp53*^*R270H*^ but not *Trp53*-null mutation promotes the metastatic potential of cancer cells.

In the present study, we also demonstrate that the nuclear localization of p53^R270H^ is required for the induction of MPAGS expression, which subsequently induces the malignant phenotypes of intestinal tumors. Moreover, recent studies have shown that a p53 gain-of-function induces a global shift in gene expression through chromatin modification,^19, 20^ and we also consistently found that the promoter accessibility of MPAGS increased. It is therefore conceivable that the remodeling of chromatin in gene promoters by nuclear localized mutant p53 causes hyper-activation of transcription factors, leading to malignant phenotypes. Because >94% of human CRCs carry genetic alterations in the Wnt signaling pathway, such as in APC and β-catenin,^3^ the TCF/β-catenin complex is one of the possible transcription factors that are activated by mutant p53 in colon cancer cells. Indeed, our study demonstrates that the canonical Wnt pathway that had already been activated in *Apc* mutant tumors was further augmented by p53^R270H^, likely through an increased accessibility of Wnt-target genes to the β-catenin/TCF complex, or via the induction of cofactors that enhances the transcription of Wnt-target genes. It has been shown that an increased Wnt/β-catenin activity in CRC cells correlates with an invasive phenotype and cancer stem cell property.^32, 33^ Thus, it is possible that the hyper-activation of the Wnt/β-catenin signaling may be a mechanism that drives the malignant progression induced by p53^R270H^.

It has been shown in 3D cultures that the activation of Wnt/β-catenin signaling together with the epidermal growth factor pathway can induce the tube-like structures of intestinal epithelial cells.^34^ Moreover, such a mechanism may increase the invasive growth of tumor glands into stromal tissues.^35^ We herein observed that p53^R270H^ induced marked morphological changes of tumor organoids to complex glandular structures that correlated with increased invasiveness. Moreover, we also demonstrated increased branching of the tumor glands in human CRC with *TP53* mutations. These data indicate that such morphological and invasive features are driven by p53 mutations, fueled by a hyper-activated Wnt/β-catenin pathway to promote invasion and metastasis (Supplementary Figure 6).

We have previously demonstrated that inflammatory responses have a role in the malignant progression of intestinal tumors.^36^ It has been reported that mutant p53 prolongs TNF-α-induced NF-κB activation, which results in highly prone to inflammation-associated colon cancer,^16^ and that mutant p53 facilitates TNF-α signaling.^37^ These results, taken together, suggest that mutant p53 has a role in malignant progression through the induction of the inflammatory pathway. We show here that inflammatory and innate immune signaling pathways are significantly activated in tumor cells by mutant p53^R270H^ expression, which may be caused by the activation of NF-κB through mutant p53-induced promoter accessibility. Accordingly, *TP53/Trp53* mutation in cancer cells can activate the inflammatory microenvironment, which contributes to malignant progression.

Importantly, it has been shown that inflammatory and innate immune pathways through NF-κB and TLR2 together with Wnt/β-catenin signaling are important for the acquisition of stem cell properties.^30, 38^ Accordingly, the present results suggest that mutant p53^R270H^ can induce stem cell properties in intestinal tumor cells through the activation of both NF-κB pathway and Wnt signaling (Figure 7b and Supplementary Figure 6). Moreover, it is possible that the cytokines expressed by cancer cells further activate inflammatory signaling in tumor cells by some positive feedback mechanism, which then accelerates mutant p53^R270H^-induced malignant progression.

In conclusion, we herein showed that *Trp53*^*R270H*^ mutation promotes the invasion and metastasis of intestinal tumors. Nuclear-accumulated mutant p53^R270H^ induces the acquisition of invasiveness associated with complex glandular structures of tumor organoids, which drives the submucosal invasion and distant metastasis of CRC. The activation of the inflammatory pathway together with Wnt signaling may be important factors to accelerate these mutant p53-induced malignant phenotypes. Therefore, the dynamic regulation of nuclear accumulation for mutant p53 represents an attractive treatment strategy against the malignant progression of CRC and thus warrants further investigation.

## Materials and methods

### Mouse experiments

*Apc*^*Δ716*^ and *villin-CreER* mice were previously described.^21, 39^ *Tgfbr2*^*flox/flox*^, *Trp53*^*LSL·R270H*^ and *Kras*^*LSL•G12D*^ mice were obtained from the Mouse Repository (NCI-Frederick, Frederick, MD, USA).^11, 40, 41^ The genetic background of all strains used in this study is C57BL/6. All animal experiments were performed with the protocol approved by the Committee on Animal Experimentation of Kanazawa University. The generation of the compound mice used in this study is provided in the Supplementary Materials and Methods. For the survival rate analysis, mice were observed until 264 days of age, and the mice were euthanized when they showed a moribund phenotype. The total numbers of polyps in both the small intestine and colon were scored and examined histologically at 22–29 weeks of age (*n*=5 each genotype).

### Human CRC samples

The human CRC tissues were collected in National Cancer Center Hospital East, Japan with informed consent and nine samples were selected after examination of the *TP53* mutation status (*n*=2 each for R273H, and R273C, *n*=1 each for V272M and P278S, *n*=3 for wild type). The four of the nine cases were collected for BREAC trial (Yuki *et al.*, ASCO, 2015; Yamazaki *et al.*, ASCO, 2015). The genetic and clinicopathological information for the human samples are presented in Supplementary Table 2. This study was approved by the institutional review board of the National Cancer Center East (registration #2005-043), and carried out according to the ethical principles of the Declaration of Helsinki.

### Histology and immunohistochemistry

The tissue specimens were fixed in 4% paraformaldehyde, paraffin-embedded and sectioned at 4*-*μm thickness. The sections were stained with Haemotoxylin and Eosin or Masson’s trichrome stain. For immunohistochemistry, antibodies against E-cadherin (R&D Systems, Minneapolis, MN, USA), α-smooth muscle actin (Sigma, St Louis, MO, USA), p53 (CM5) (Leica Biosystems, Wetzlar, Germany), Snail2 (NOVUS Biologicals, Littleton, CO, USA), mesothelin (IBL, Fujioka, Japan), and Ki67 (Life Technologies, Grand Island, NY, USA) were used. Staining signals were visualized using the Vectastain Elite Kit (Vector Laboratories, Burlingame, CA, USA). Human CRC histology sections were also purchased (US Biomax, Rockville, MD, USA).

### Scoring tumor gland branching

The branching frequency of the mouse tumor glands was scored using Haemotoxylin and Eosin sections (*n*=4~6 mice for each genotype). The branching numbers of the individual continuous glands found on the sections were scored blindly in two randomly selected microscopy fields, and three polyps were examined per mouse. The ratio of the numbers of branches was then calculated. The branching frequency of human CRC was scored blindly using Haemotoxylin and Eosin sections. The number of gland branching was counted in three individual glands at the invasion front, and the mean values were calculated.

### Cell culture experiments

The mouse intestinal tumor cell line (AP-MM6) was established using *Apc*^*Δ716*^ *Trp53*^*R270H/R270H*^ intestinal tumor-derived organoids by subcloning in a 96-well plate (deposited to Riken BioResource Center, Tsukuba, Japan). p53 expression vectors were transfected to AP-MM6 cells using Lipofectamine 2000 (Thermo Fisher Scientific, Rockford, IL, USA), and examined by immunocytochemistry using antibody for p53 (Leica Biosystems), E-cadherin (R&D Systems) and anti-rabbit IgG Alexa 488 as a secondary antibody (Molecular Probes, Eugene, OR, USA). The constructions of the p53 expression vectors are provided in the Supplementary Materials and Methods. The ratio of cells with cytoplasmic p53 was calculated by counting>4 × 10^3^ cells, and experiments for each transfection condition were performed three times independently. The staining intensity of nuclear p53 was measured using the NIH image software program (NIH, Bethesda, MD, USA).

### Organoid culture experiments

The organoid cultures were prepared from small intestinal tumors, as previously described.^42^ Organoid cell proliferation was examined using the Click-iT EdU Imaging System (Invitrogen, Carlsbad, CA, USA), and the EdU labeling index was calculated by counting more than 200 cells each for three independent organoids. The organoids were immunostained using anti-p53 antibody (Leica Biosystems) and Alexa Fluor 488-conjugated antibody (Molecular probes, Grand Island, NY, USA). For the inhibition of p53^R270H^, organoids were treated with pan-histone deacetylase inhibitor suberoylanilide hydroxamic acid (Sigma). The organoid morphologies were categorized as cyst, simple tube or complex tube structures based on their morphologies (Supplementary Figure 4a).

For the invasion assay, the organoids were dissociated with Accutase (Innovative Cell Technologies, San Diego, CA, USA), and 3 × 10^4^ cells in Matrigel were seeded in Fluoroblock inserts with 8-μm pores (Corning, Corning, NY, USA). The culture media of the upper and bottom wells were the same as the organoid culture media. At 10 days after seeding, the cells invading through the pores of the inserts were stained with fluorescent dye Calcein AM (Corning) and examined with a fluorescence microscope. When invading cells were found, the well was judged to be ‘invasion-positive’, and the total number of ‘invasion-positive’ wells was scored for each genotype.

For the suspension array analysis, 1.5 × 10^5^ cells of organoids were cultured for 4 days and the culture medium was collected and used for suspension array analyses according to the manufacturer’s instructions of the Bio-Plex Mouse Cytokine Assay kit (Bio-Rad, Hercules, CA, USA).

### Organoid transplantation experiments

NOD/Shi-*scid Il2rg*−/− mice (NOG mice) were purchased (CIEA, Kawasaki, Japan). The organoids were mechanically dissociated, and 1 × 10^5^ organoid cells were injected s.c. with Matrigel to NOG mice (*n*=4–7 dependent on genotypes). At 4 months after transplantation, the tumors were examined histologically. For the liver metastasis analysis, 1 × 10^5^ organoid cells were injected with Matrigel into the NOG mouse spleens (*n*=2–4 dependent on genotypes). At 1 month after injection, the liver metastases were examined histologically.

### Real-time RT-PCR

For real-time RT-PCR, tumor cells from the invasion and non-invasion areas of intestinal tumors of mice were isolated from frozen sections on a LMD7000 laser microdissection system (Leica Microsystems, Wetzlar, Germany). Total RNA was extracted from laser microdissection samples using an RNeasy Plus Micro extraction kit (Qiagen GmbH, Hilden, Germany), reverse transcribed using a PrimeScript RT reagent kit (Takara, Tokyo, Japan) and amplified using ExTaqII SYBR Premix (Takara) on a Stratagene Mx3000P real-time thermocycler (Agilent Technologies, Santa Clara, CA, Japan). The primer sequences are described in the Supplementary Materials and Methods.

### Next-generation RNA sequencing

Poly A(+) mRNA extracted from the organoids (*n*=2 for each genotype) was used for the sequencing library construction by a TruSeq Stranded mRNA LT Sample Prep Kit (Illumina, San Diego, CA, USA) and an Agilent XT Auto System (Agilent Technologies). The libraries were sequenced by an Illumina HiSeq 2500 (Illumina). The raw reads were mapped to the mouse reference genome (mm10), and the reads per kilobase per million mapped reads (rpkm) values of the genes were calculated using the StrandNGS software package (Strand Genomics, San Francisco, CA, USA). The sequencing data were deposited in the Gene Expression Omnibus (accession code GSE81441).

### Upstream pathway and gene ontology analyses

The MPAGS was analyzed for putative upstream regulators and processes using the Ingenuity Pathway Analysis and gene ontology Analysis software package (Ingenuity Systems; www.ingenuity.com). The activation *z*-scores were calculated as a measurement of the functional and translational activation in the upstream regulator analysis. Pathways with *z*-scores of >2 and *P*-values of <0.05 were designated as activated with statistical significance.

### Statistical analysis

The data were analyzed using an unpaired *t*-test and are presented as the means±s.d. A value of *P*<0.05 was considered to be statistically significant.
