# Supplementary material for: Intestinal cancer progression by mutant p53 through the acquisition of invasiveness associated with complex glandular formation
Source: Oncogene. 2017 Jun 19;36(42):5885–96. doi: 10.1038/onc.2017.194 (PMC5658682; doi:10.1038/onc.2017.194)
Supplement: Supplementary Materials and Methods [file onc2017194x1.doc]

**Supplementary Materials and Methods**

*Generation of compound mouse strains*

To generate *Apc^∆716^ Trp53^R270H/R270H^* and *Apc^∆716^ Trp53^+/R270H^* mice, *Apc^∆716^ Trp53^LSL·R270H/LSL·R270H^ villin-CreER* and *Apc^∆716^ Trp53^+/LSL·R270H^ villin-CreER* compound mutant mice were treated with tamoxifen at 4 mg/mouse once a week from 8 weeks of age. *Apc^∆716^ Kras^G12D^*, *Apc^∆716^ Tgfbr2^∆IEC^*, and *Apc^∆716^ Kras^G12D^ Trp53^+/R270H^* mice were generated by treating *Apc^∆716^ Kras^LSL·G12D^ villin-CreER*, *Apc^∆716^ Tgfbr2^flox/flox^ villin-CreER*, and *Apc^∆716^ Kras^LSL·G12D^ Trp53^+/LSL·R270H^ villin-CreER* compound mice with tamoxifen, respectively, at 4 mg/mouse once a week from 8 weeks of age.

*Histology and immunohistochemistry*

For immunohistochemistry of Ki67, anti-Ki67 antibody (Life Technologies, Grand Island, NY, USA) was used. The mean Ki67 labeling index was calculated as the number of Ki67 positive cells divided by the total number of cells by counting 5 independent microscopic fields (× 200). For apoptosis analysis, the ApopTag *In situ* Apoptosis Detection Kit (Millipore, Darmstadt, Germany) was used. For immunohistochemistry of human p53, anti-human p53 antibody (DO-7) (DAKO, Santa Clara, CA, USA) was used as the primary antibody.

*Construction of wild-type p53 expression vectors*

For the construction of the p53 expression plasmid, the coding region of wild-type murine p53 cDNA was amplified by RT-PCR from C57BL/6 intestinal RNA with the primers: CCAGCAGGGTGTCACGCTTCT (forward) and GATGCAGAGGCAGTCAGTCTGAGTCA (reverse). The amplified p53 cDNA was subcloned to a pcDNA 3.1(+) expression vector (Invitrogen, Carlsbad, CA, USA) to construct wild-type p53 expression vector, and the sequence was confirmed.

*Primer sequences for RT-PCR.*

The primer sequences used for RT-PCR are as follows (purchased from Takara).

| Gene | Forward | Reverse |
| --- | --- | --- |
| *p53* | AACTTACCAGGGCAACTATGGCTTC | AACTGCACAGGGCACGTCTTC |
| *Cd44* | TTTAACCTATATGCAGCAAGCCACT | CAGAATCATCACCACTATGGCAAG |
| *Hoxa10* | AGCAAAGCGCCTTCGTTAG | AGCGTCTGGTGCTTCGTGTA |
| *Gata2* | GGCTCTACCACAAGATGAATGGA | CGCCATAAGGTGGTGGTTGTC |
| *Cxcl5* | TGATCCCTGCAGGTCCACA | CTGCGAGTGCATTCCGCTTA |
| *Fzd10* | GCCACCCAGCACAAGTGTAAGA | CCAGCAGCATGGACACTTTGA |
| *Hoxa9* | AGAATGAGAGCGGCGGAGAC | AGCGAGCATGTAGCCAGTTG |
| *Sox11* | AAGCATGGTGATCGTGTCTTGG | ATGGAGTTTGCTGGCATTGGA |
| *Lef1* | CAACCAGATCCTGGGCAGAAG | CGCTGACCAGCCTGGATAAAG |
| *Wnt5b* | CTCTCATGAACCTACAGAACAACGA | TGGAGCCAGCAGGTCTTGA |

*Clustering analysis*

For clustering analyses of the RNA sequencing data generated from *Apc^∆716^ Trp53^+/+^*, *Apc^∆716^ Trp53^flox/flox^*, and *Apc^∆716^ Trp53^R270H/R270H^* organoids, low-expressing genes that showed less than 4 reads per kilobase per million mapped reads (rpkm) in all 3 genotype samples were first removed. Following this pre-processing, average-linkage clustering analyses with correlation-based metric were performed with the Cluster 3.0 software package,^1^ and the clustering results were visualized using the Java TreeView software program.^2^

*Chromatin accessibility assay*

*The chromatin accessibility of the promoter regions was ascertained using a Chromatin Accessibility Assay Kit (Abcam, Cambridge, UK). The isolated chromatin DNA complexes from organoid cells were untreated or treated with nuclease before DNA extraction and purification. A genomic PCR for the promoter regions of the respective genes was then performed. The primer sequences used for this study are as follows. The fold enrichment (FE) was calculated as the ratio of the amplification efficiency of the nuclease-treated DNA samples to that of the undigested control counterparts, using the following formula: FE = 2 ^(Nse Ct – noNse Ct)^ × 100(%)*

| Gene | Forward | Reverse |
| --- | --- | --- |
| *Hoxa9* | ACTCTCAGTTGCCGCTGTTT | TGGAAGCGGAGAGACAGATT |
| *Hoxa10* | GCTCAAAGGAGAGCGAGAGA | CGCATACTCCCCATCCTCTA |
| *Gata2* | AGAAGCCGAGAAAGCCTTCC | GGGAGGTCTAGCAAGAGGGA |
| *Lef1* | ACCCTATCACACTGGAGGCT | ACAACAAGGGGCTGGTGAAA |
| *Sox11* | ACTCGCGGATTTCTTTTGAA | CACTACTCCCACCAGCCAAT |
| *Fzd10* | AAGGGTCACCACGATTCAAG | AAATTTCAAAAGGGCCGAGT |
| *Cxcl5* | ACATGGCCAGAGGTTTTGAG | GCCACCTGGTGATGAAAGAT |
| *Wnt5b* | GGACTCCCCAAAGCCTCTTC | TGTTTGGCCTTAGCTCAGGG |

References

1. de Hoon MJL, Imoto S, Nolan J, Miyano S. Open source clustering software. *Bioinformatics* 2004; **20**: 1453-1454.

2. Saldanha AJ. Java Treeview-extensible visualization of microarray data. *Bioinformatics* 2004; **20**: 3246-3248.
