# Supplementary figures and images for: Intestinal cancer progression by mutant p53 through the acquisition of invasiveness associated with complex glandular formation
Source: Oncogene. 2017 Jun 19;36(42):5885–96. doi: 10.1038/onc.2017.194 (PMC5658682; doi:10.1038/onc.2017.194)

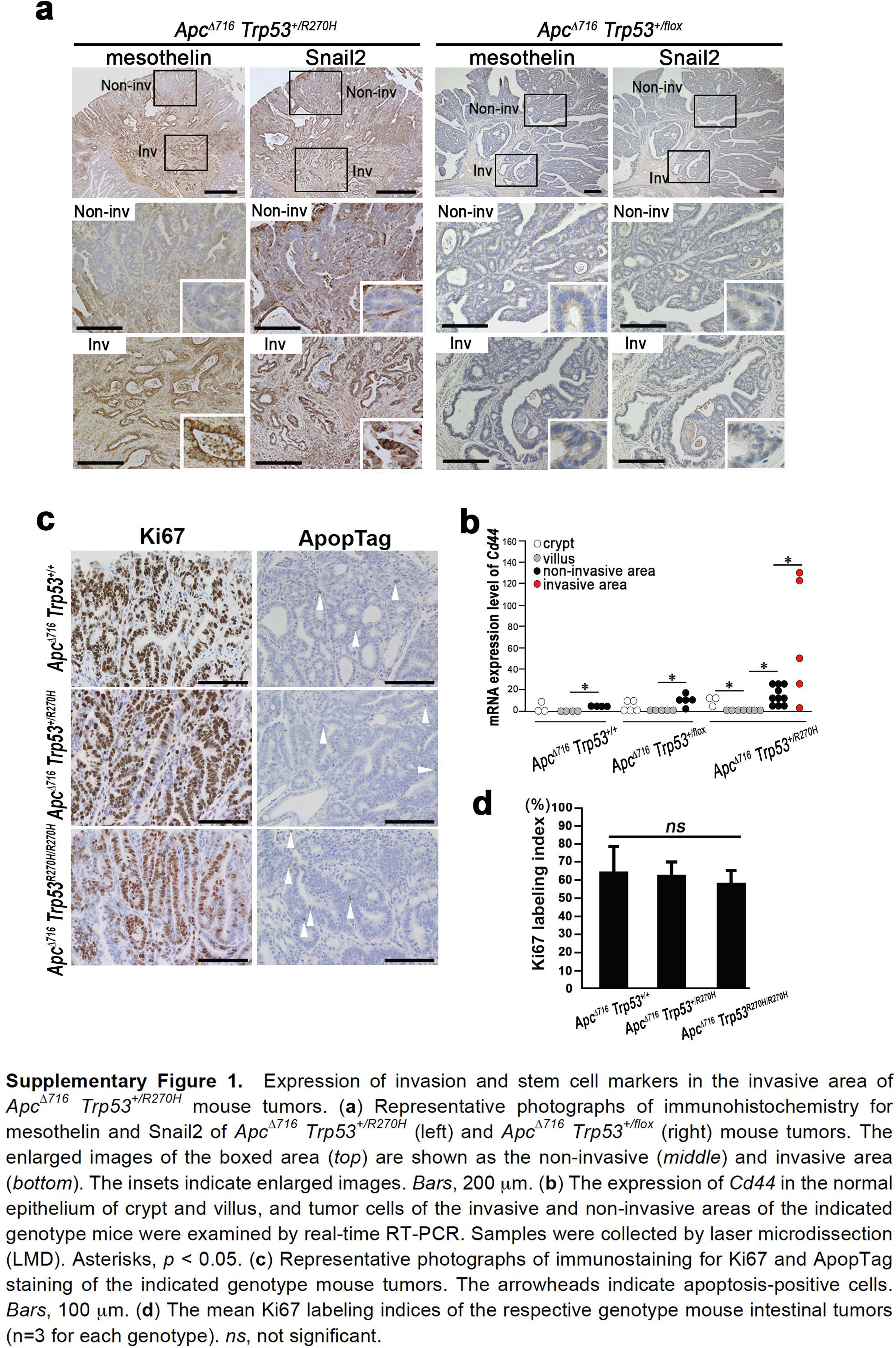

Supplement: Supplementary Figure 1 [file onc2017194x2.tif]

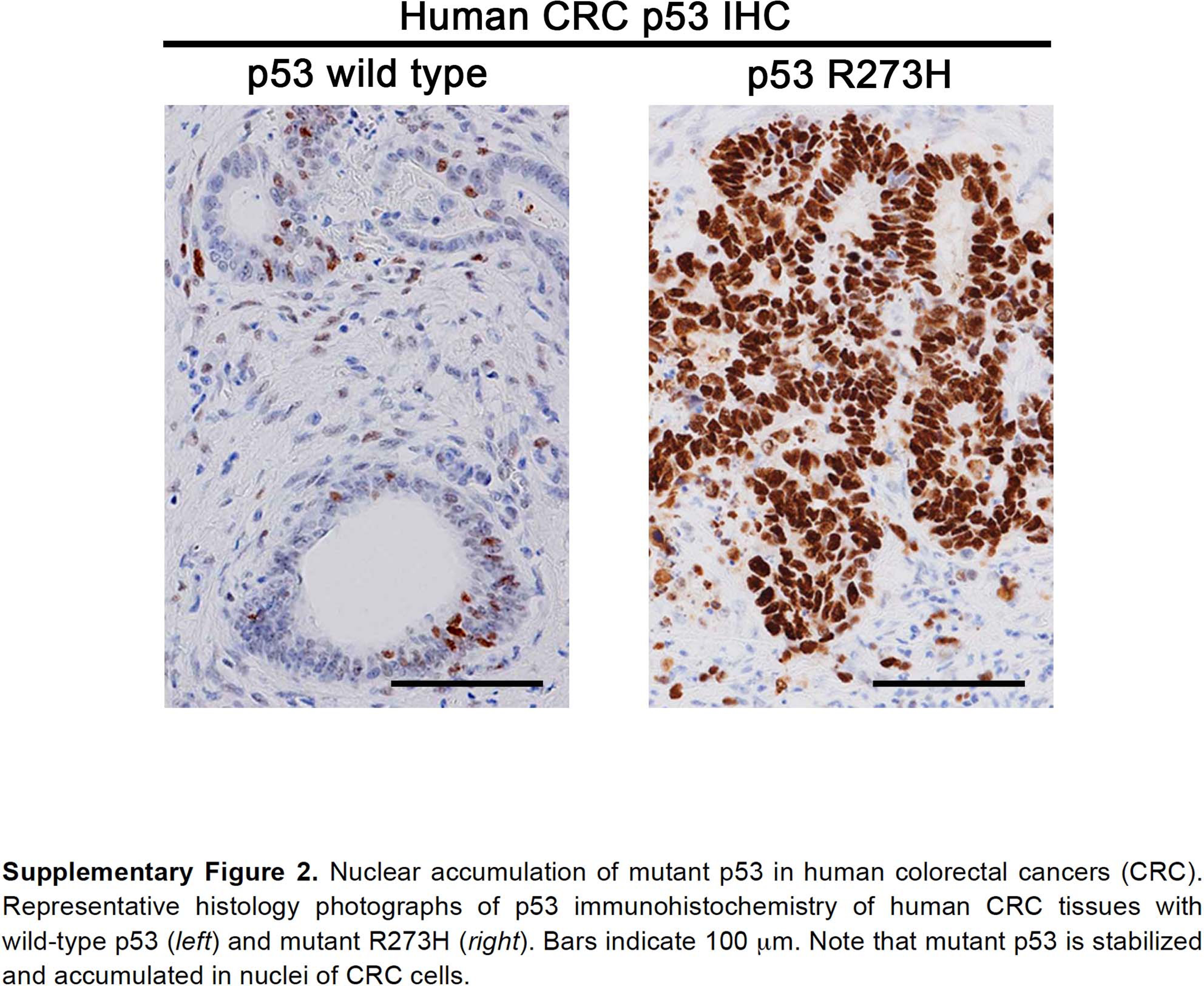

Supplement: Supplementary Figure 2 [file onc2017194x3.tif]

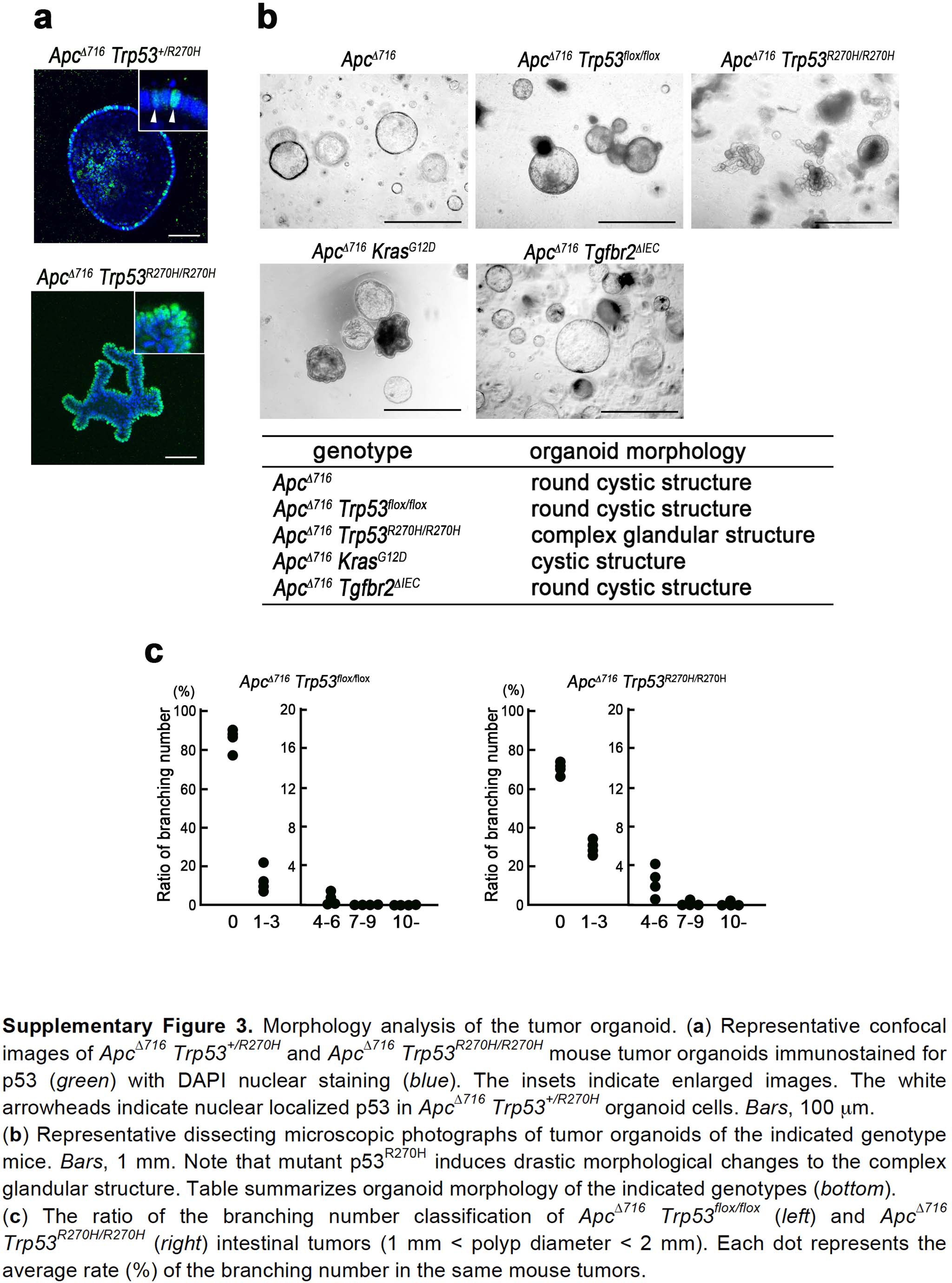

Supplement: Supplementary Figure 3 [file onc2017194x4.tif]

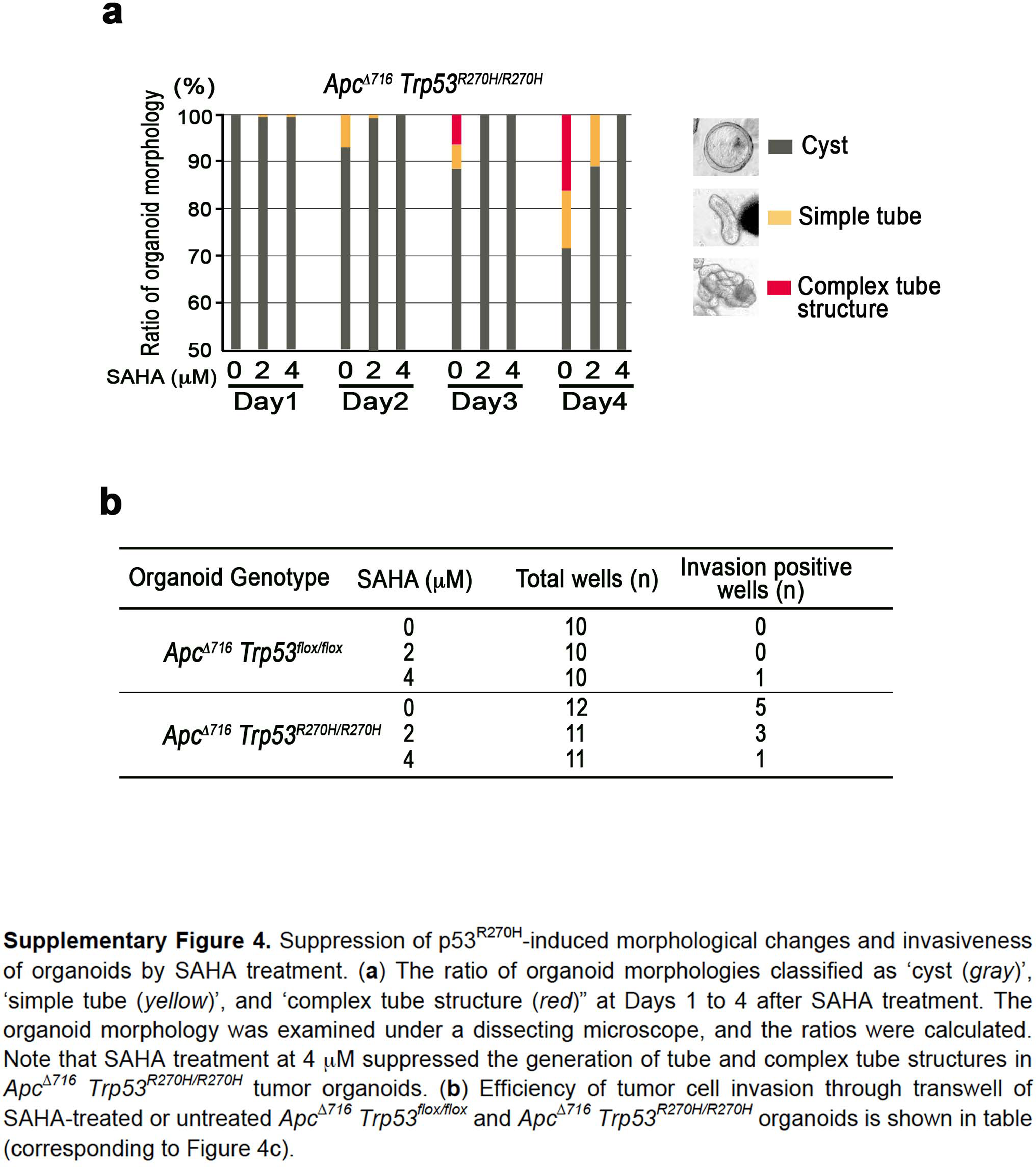

Supplement: Supplementary Figure 4 [file onc2017194x5.tif]

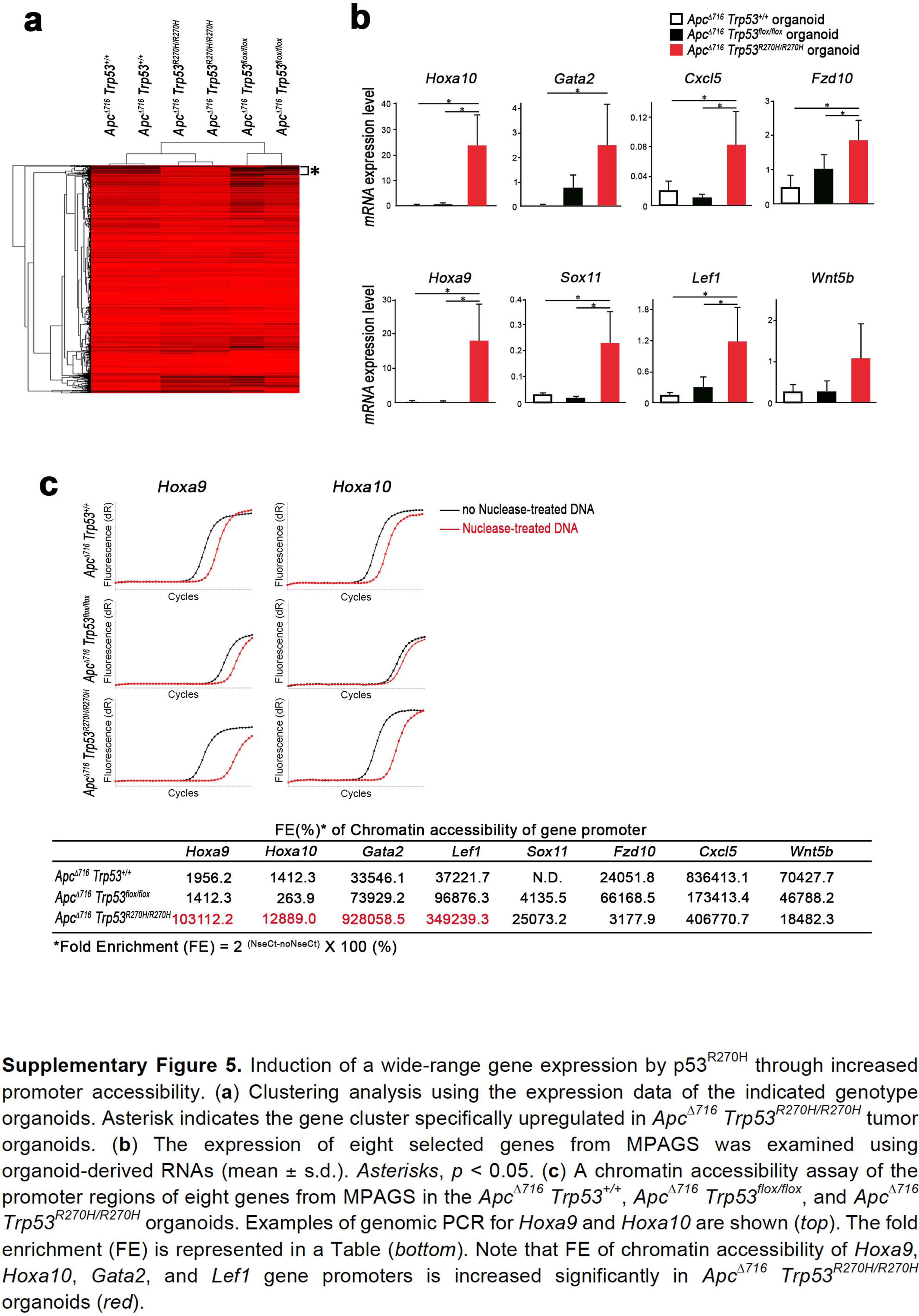

Supplement: Supplementary Figure 5 [file onc2017194x6.tif]

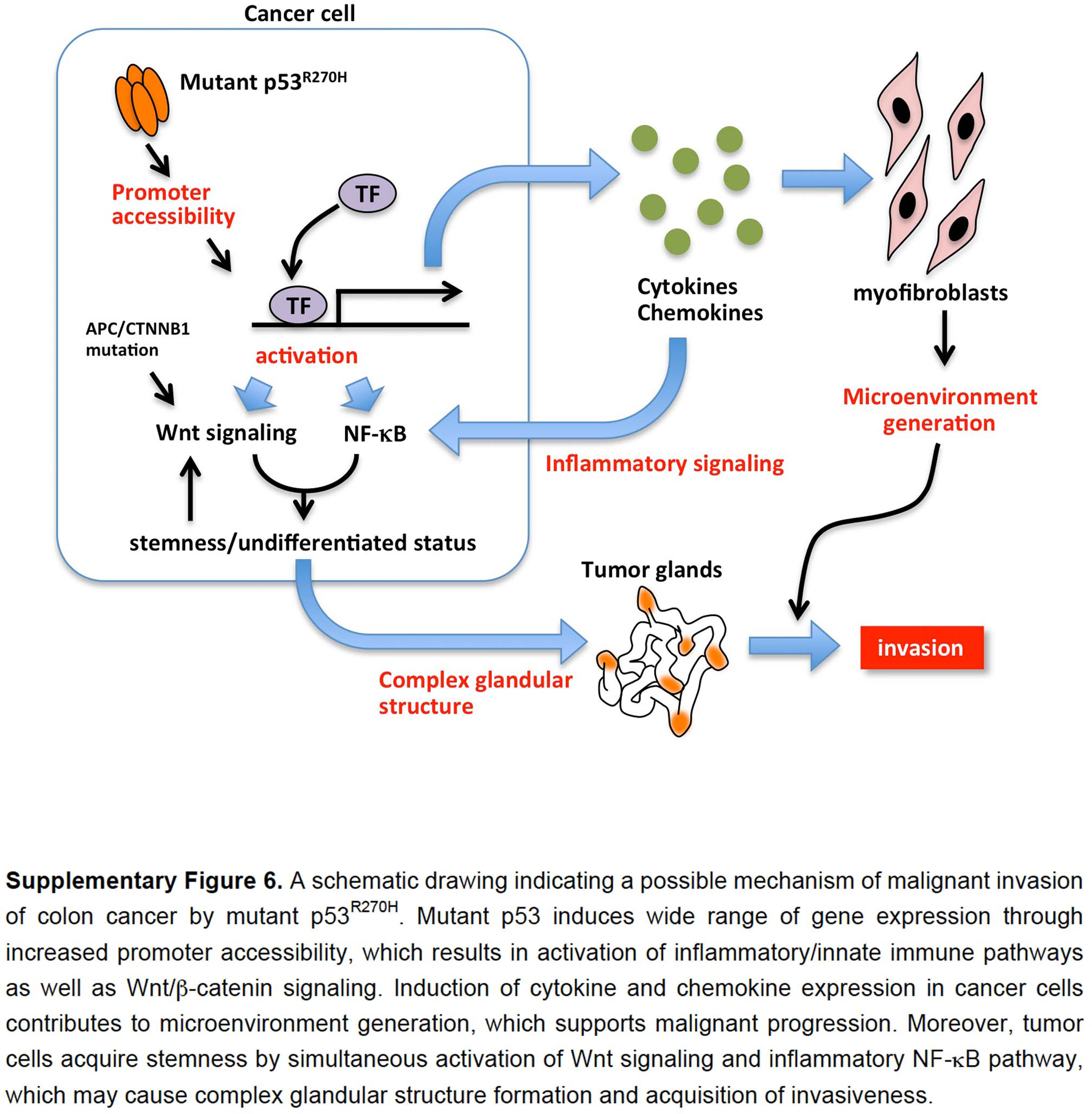

Supplement: Supplementary Figure 6 [file onc2017194x7.tif]
