# Supplementary material for: Intestinal cancer progression by mutant p53 through the acquisition of invasiveness associated with complex glandular formation
Source: Oncogene. 2017 Jun 19;36(42):5885–96. doi: 10.1038/onc.2017.194 (PMC5658682; doi:10.1038/onc.2017.194)
Supplement: Supplementary Table 1 [file onc2017194x8.pdf]

Supplementary Table 1. List of "the mutant p53-activated gene set (MPAGS)" .

			Reads per kilobase per million mapped reads (rpkm)						Fold change vs wild type (mean WT)	
Gene ID	Gene Symbol[a]	Entrez ID	WT1[b]	WT2	p53(M/M)1	p53(M/M)2	p53(flox/flox)1	p53(flox/flox)2	p53(M/M) vs WT	p53(flox/flox) vs WT
14955	H19	14955	1.88718081	1.90717677	117.630356	139.987034	1.630273222	2.881873281	67.89486357	1.189172715
109294	Gm1123	382097	3.83573752	3.39673036	59.7738474	53.8841327	7.65095533	1.042454123	15.71496506	1.201997657
613254	Tubb4a	22153	4.09259583	4.01926778	61.8241888	64.9572882	1	9.60148929	15.62914307	1.306911678
11536	Podn	242608	1.44788087	1.26958404	18.2204742	17.6276188	1.223057278	1	13.19174091	0.81806292
22269	5730457N03Rik	70593	1	1	14.0131171	10.9544899	1	1	12.48380349	1
13609	Rfx4	71137	1	1	12.573222	11.6681117	1	1	12.12066688	1
18053	Apcdd1	494504	23.1540715	18.5996749	244.422892	238.362091	31.65345337	3.842507564	11.56267461	0.850126372
380924	Hmgcs2	15360	2.89412754	2.62146594	29.0192032	30.0116139	1	2.865683937	10.70253226	0.700864549
22409	Efcab1	66793	1	1	10.5512923	10.530491	1	1	10.54089164	1
16324	Oas1a	246730	1.00311625	1.14059699	9.85301463	12.3855549	1	3.27725311	10.37385464	1.995254324
83410	Slc30a2	230810	18.0570161	14.4709477	163.413855	172.480685	18.32127499	22.88031485	10.32633161	1.266651371
65969	Mt3	17751	1.38436008	1.26817881	13.4023121	13.008132	1	1.543913109	9.956666123	0.959048374
380712	Hoxa10	15395	1	1	10.143414	9.72258943	1	1	9.933001711	1
24110	Ngfr	18053	1	1	10.5616842	9.26860232	1	1	9.91514324	1
18946	Fam70a	245386	1	1	9.97261393	9.28035954	1	1	9.626486736	1
15395	Spock2	94214	9.66977821	6.89395891	75.8774873	79.0233115	1	10.16167977	9.35180254	0.673862407
240638	Slc16a12	240638	1.48462236	1.3608142	14.2949582	12.3111156	1	1	9.350436512	0.702879843
1E+08	Prex2	109294	1	1	9.77917459	7.78863224	1	1	8.783903418	1
18822	Mira	1.01E+08	1	1	10.0679545	7.3784409	1	1.563284764	8.723197712	1.281642382
64706	Upk2	22269	1	1	8.35958718	9.07199472	1	1.30474828	8.715790949	1.15237414
22153	Htra1	56213	2.71436381	2.81301491	26.4004442	20.8807409	1	1.653966025	8.553997752	0.480149119
230085	S1pr1	13609	1	1	8.78311078	8.07842138	1	1	8.430766077	1
18816	Afp	11576	1	1	8.41933858	7.72873607	1	1	8.074037324	1
72190	Atp10a	11982	1.9454736	1.99996423	17.619394	14.0229327	1	2.405605714	8.019978545	0.863175612
26971	Pcdh7	54216	1.33031952	1.21308183	10.4024323	9.77555627	1	1	7.933466189	0.786348564
18616	Vdr	22337	3.43093097	3.58538936	26.5306662	27.7585692	5.749309114	2.871294478	7.737565108	1.22865023
56811	Tle6	114606	1.60588584	1.45952225	12.1935576	11.1320239	1.259503246	1.600834983	7.609290753	0.933101938
13601	Cubn	65969	1.20819222	1.39317713	10.3031387	9.06857268	3.754908103	1	7.446736232	1.827848128
93897	Pla2g2f	26971	14.7764489	13.2109591	102.679616	104.816316	14.84556813	8.786722915	7.41390315	0.844390128
101602	9330117O12	328957	1	1	7.71459275	6.91865499	1	1	7.31662387	1
70571	Usp18	24110	1.14147997	1.55304489	11.0017003	8.66813847	1	1	7.299928485	0.74224589
50934	Tcerg1l	70571	1	1	7.92310214	6.64851436	1	1	7.285808248	1
20840	Lrrn1	16979	1	1	7.67885178	6.82557128	1	1	7.252211527	1
22270	Otop1	21906	1	1	7.35580431	7.09295699	1	1	7.224380652	1
17751	Dlx3	13393	2.10017464	2.22710534	14.938642	15.655867	1.186801553	1	7.070147787	0.505352453
382097	2510009E07Rik	72190	4.06718721	3.66639774	29.4534045	24.9932637	1	1.905654	7.040288375	0.375718896
21380	Gata2	14461	1	1	6.85580607	7.20584141	1	1.983907374	7.030823737	1.491953687
73916	Lmo2	16909	1	1	7.10218431	6.92041953	1	1	7.01130192	1
665927	Cxcl5	20311	1	1	7.14290533	6.80040185	1	2.526455029	6.971653587	1.763227514
12405	Tmem59l	67937	1	1	6.86739049	6.67686097	1	1	6.772125731	1
245297	Gm15308	1E+08	1	1	8.15199801	5.36435048	1.351008058	1	6.758174249	1.175504029
13393	Fzd10	93897	2.88072092	2.66158972	18.8738783	18.2753648	2.820139249	1.159064643	6.702843907	0.717968398
70593	Nptx1	18164	3.31436433	3.2542762	21.189495	21.4579091	1	1.530665636	6.492576955	0.385264748
67086	N28178	230085	1	1	7.0705792	5.90980693	1	1	6.490193064	1
69047	Rnf152	320311	1.43422031	1.22747803	9.40810655	7.84642075	1	1	6.482525492	0.751399952
320311	Trim62	67525	1.50381091	1.54009104	10.0082012	9.64186948	1	1.360295184	6.455553116	0.775417613
12560	Ism1	319909	1.1712029	1.1719861	8.85178862	6.26699786	1	1	6.452226636	0.853537636
230810	Dkk2	56811	1.93842424	2.65580606	15.1654395	14.4156821	1	1	6.438754623	0.435328634
11551	Cstf2t	83410	1.83190501	1.86390543	11.6871901	11.5289642	1	5.487647071	6.281749201	1.755405797
244654	Morn4	226123	1.20518385	1.19853223	7.53760608	7.54949268	1	3.052500222	6.276572714	1.68593132
50795	Serpinf2	18816	1	1	5.41519616	7.1037982	1	1	6.259497184	1
71137	Zim1	22776	2.627829	2.80920654	17.9580202	15.6471218	1	1	6.180783938	0.367847512
17380	Sema3e	20349	1	1	6.40161671	5.88573587	1.430292982	1	6.143676289	1.215146491
1E+08	Scube1	64706	2.179316	2.07177697	12.8696356	13.055652	1	1	6.098499329	0.470467246
627821	Cdh3	12560	1	1	6.35840979	5.80959986	1	1.205385454	6.084004826	1.102692727
21413	Lbp	16803	1	1.14452613	7.31428479	5.73266629	1	1	6.083838709	0.932606963
218203	Bpifb1	228801	1	1	6.36680686	5.75062595	1	1	6.058716404	1
1E+08	Bpifb5	228802	7.27510666	9.00338741	47.6509906	50.7734715	1	1.896325433	6.046287921	0.177923426
18106	Slc7a8	50934	1	1	6.72743478	5.27542454	1	1	6.001429662	1
103142	Stac	20840	1	1	5.59994283	6.30530824	1.376255625	1	5.952625538	1.188127812
11576	Upk3a	22270	6.77252869	6.149621	33.1303146	43.4536723	3.856053585	2.788057084	5.926567079	0.514164503
229521	Hoxa9	15405	1	1	6.41686219	5.23476401	1	1	5.8258131	1
11303	Evx1	14028	1	1	6.09822339	5.52724897	1	1	5.812736182	1
242484	Cacna1a	12286	1.00302541	1	5.31995189	6.20210951	1	1	5.752329118	0.998489579
72090	Ift57	73916	1.09749055	1.16687704	6.58084435	6.39932616	2.457745445	1.928851398	5.732360144	1.937228243
228801	Dennd2a	209773	1.2445494	1.39513529	7.68656486	7.23195449	1	1	5.651629304	0.757666249
18761	Prss56	69453	1	1	4.71796724	6.56820839	1	1	5.643087813	1
22041	5930412G12Rik	319616	1	1	5.66836555	5.6159224	1	1	5.642143974	1
12159	Plod1	18822	1.06951809	1.09552861	5.61798199	6.32914403	1.132758259	1.607837428	5.518183977	1.265836752
170776	Cbln2	12405	1	1	6.29295445	4.56346827	1	1	5.428211359	1
225266	4921525O09Rik	74050	1	1	5.89824807	4.9197377	1	1	5.408992885	1
20198	Comp	12845	1	1	5.28279051	5.45036104	1	1	5.366575775	1
232664	4933436C20Rik	71296	1.07853127	1.08064318	5.55534766	5.95360456	2.519418347	1	5.33025585	1.629983327
15160	Tcf4	21413	6.89282554	6.53818183	37.9285049	33.3344727	9.736544158	5.750830435	5.305855002	1.153105956
53412	1700020N18Rik	67086	1	1	4.99999066	5.55652648	1	1	5.278258568	1
12845	Esyt3	272636	1.17983043	1.42915738	7.49339707	6.16925295	1	1.048591256	5.23676267	0.785205374
15007	Svopl	320590	1	1	5.38565382	5.05006445	1	1	5.217859137	1
30939	Irx5	54352	3.8963876	3.98387201	18.868973	21.9697873	8.290878054	1	5.182413064	1.179006595
69354	Peg3	18616	32.1555665	34.2242732	182.17052	158.01857	1	3.660130784	5.124885678	0.070204008
192201	Nxpe4	244853	1	1	5.22500095	4.99822033	2.380128558	1	5.111610642	1.690064279
194126	Pik3ap1	83490	1.341897	1.21621668	6.30715986	6.70770667	1	1.74695446	5.087681062	1.073820323
23960	Mylip	218203	1	1	5.18291452	4.80292407	1	1.005262494	4.992919293	1.002631247
14461	Agmat	75986	1.54762029	2.11086542	9.03969627	9.20930829	1	1	4.988130613	0.546674269
211135	Cd244	18106	1.36293817	1.26096271	7.3473281	5.73675657	1.147693754	1.860421284	4.98650111	1.146428608
320590	Vim	22352	68.9606276	49.9526219	306.032487	284.012969	71.57029395	19.83942076	4.961982437	0.768709248
19662	Syt11	229521	2.38156149	2.04346764	11.9457175	10.0072634	1.350090137	2.636022163	4.96109295	0.900810407

15405	Mt4	17752	1.5238582	1.41778192	6.2433563	7.88611255	1	1	4.803262215	0.679892821
94214	Abca1	11303	3.05583998	2.96995197	16.1748116	12.7045133	1	1.169082766	4.792618972	0.359966421
83490	D630039A03Rik	242484	10.9263634	10.9403971	55.0349657	49.2895874	9.383700711	7.248233841	4.770919464	0.760603501
382059	Gm15340	1E+08	1	1	4.84112149	4.6507842	1	1	4.745952847	1
23966	Prkcq	18761	3.28167371	3.53956409	17.3796365	14.8954056	4.572492489	2.160541586	4.731552099	0.987069249
72709	Trf	22041	2.04741875	2.13212571	9.73365338	10.0100391	1	3.549045327	4.723886216	1.088406972
12552	Sp6	83395	1.03462636	1.12401068	5.34056952	4.78878146	1	1	4.692475277	0.92651055
12577	Gm6792	627821	1	1	5.54466616	3.79653577	1	1	4.670600965	1
320343	Rbp4	19662	1	1	4.37426311	4.95854793	1	2.865610046	4.666405517	1.932805023
73713	Klhl14	225266	1	1	4.7732371	4.47840903	1	1	4.625823062	1
65255	S100a4	20198	2.00429005	2.47822246	11.7319604	8.95998303	3.303065778	2.158311204	4.616148505	1.218374064
54352	Ppp1r3c	53412	1.57449889	1.2270081	7.22189628	5.65434456	1	1	4.596183732	0.713901486
52808	H2-Q10	15007	4.36282166	4.08910866	17.7849498	21.0222473	8.657956188	7.62698467	4.591518827	1.926771785
15016	Inhbb	16324	1	1	4.77563707	4.39460379	1	1	4.585120427	1
16832	Otc	18416	1.17437029	1.38376378	6.12214409	5.57513987	2.519645205	1	4.572584421	1.375864251
21983	Tmod2	50876	1	1	5.08746759	4.05015185	1	1.101621148	4.568809724	1.050810574
22337	Glul	14645	71.869712	76.5320426	347.748143	323.730167	19.74380985	40.61831536	4.524732956	0.406748056
16842	Cdh11	12552	1	1	4.64852366	4.37387139	1	1	4.511197527	1
16841	Mme	17380	1	1	4.59422782	4.39523133	1	2.268440785	4.494729577	1.634220392
14028	Dkk3	50781	12.0892094	11.5425102	52.1774054	52.8959001	1.466418366	13.48590561	4.446282671	0.632722638
235604	Gpx3	14778	1	1	4.69349265	4.18136968	1	1.169332744	4.437431166	1.084666372
18416	Rdh9	103142	7.43526262	6.67785608	30.9446264	31.6166915	7.012568703	5.021960217	4.432848562	0.852719316
15360	Wif1	24117	15.4560643	16.4557103	71.3608354	69.878106	1.6569049	7.40671487	4.42591936	0.284021176
213391	Oas1g	23960	1	1	3.82761812	4.94116181	1	1.173095707	4.384389963	1.086547853
30045	Sox18	20672	1	1	4.0766856	4.63894455	1	1	4.357815075	1
1E+08	Sox11	20666	1	1	4.57361079	4.12166543	1	1	4.34763811	1
327987	Tenm4	23966	1.18373954	1	4.94115119	4.53493603	1	2.15550155	4.339385278	1.444999044
67701	Defa22	382059	11.6422105	9.49629663	46.6630434	44.7929693	3.228931197	3.208798507	4.326512383	0.304549875
246730	Tbx1	21380	3.65263096	4.18732065	17.1039105	16.5359554	1	1.251816034	4.290825701	0.287223206
22379	Cdkn1c	12577	6.41609276	4.87424612	23.530918	24.7615496	5.694010684	9.789391595	4.277326674	1.371385079
19085	Cps1	227231	1.0689553	1	3.81050709	4.97032331	1	1	4.24408898	0.966671439
22419	Rbm20	73713	1	1	4.46219945	4.02347202	1	1	4.242835735	1
16543	Serpind1	15160	1	1	4.9070154	3.48289505	1	1	4.194955225	1
108176	H2-Q5	15016	1.29512298	1.83234263	5.53299185	7.5463902	1	2.454495973	4.182102594	1.104567212
23882	Scn2b	72821	3.23679591	2.71695713	11.8329395	13.0458948	7.055197649	1.676245664	4.178680941	1.466544424
68521	Ubxn10	212190	1.04263985	1	3.91602561	4.61265375	1	1.408860335	4.17532212	1.179287838
52589	Lef1	16842	1.51787876	1.29595758	5.93274428	5.71079343	1	1	4.137958392	0.710773391
24117	Eml1	68519	2.19298873	2.27896363	9.45427612	9.04317887	1	2.708837593	4.136326484	0.829355345
27273	Fgf3	14174	1	1	3.46431952	4.75912451	1	1	4.111722018	1
1E+08	Maf	17132	2.16147957	2.98219666	11.2381979	9.68922987	1	1	4.068574066	0.388826961
15958	Prkar1b	19085	2.29388203	2.47733333	9.34635542	10.0207779	1	1.244881397	4.059161413	0.470505149
68616	Bmp4	12159	4.21536571	3.98303401	17.6155791	15.6018089	1	1	4.051691685	0.243950047
66298	Dnajc12	30045	3.41408813	2.65539485	12.3365792	12.0208558	2.807424646	3.790337198	4.013098817	1.087038529
26382	D130040H23Rik	211135	1	1	4.18119057	3.83398502	1	1.994937433	4.007587795	1.497468716
277692	Isg15	1E+08	1	1.42188738	4.98308195	4.67589619	1.362624073	2.178799438	3.988202846	1.462257718
224405	C1qtnf6	72709	1.54753217	1.57143256	6.14728682	6.28849594	1	2.41234609	3.987150812	1.094063699
625286	Reg4	67709	28.2616345	22.1148017	97.5637969	101.926615	31.06575044	35.89578144	3.959994526	1.329223283
54612	Npl	74091	2.98794108	3.13056379	13.3009981	10.7295629	1	1	3.927521749	0.326877242
22420	Wnt5b	22419	1	1	3.73264964	4.1027419	1	1	3.917695774	1
218952	Gm14446	667373	1.05013668	1	4.01179031	4.01849002	2.252378524	1.553321001	3.916948753	1.856315022
21808	Mdfic	16543	1.02642334	1.30086577	4.95315133	4.15097315	3.307121386	1.161956548	3.911900966	1.920293415
212943	Tceal8	66684	2.84404856	3.24611363	12.5849563	11.1936761	1	9.609494892	3.904433351	1.742070993
383619	Asprv1	67855	1.33431791	2.15858604	6.24505596	7.33679869	1	3.393159859	3.888413433	1.257738523
16981	Pdk4	27273	9.76883824	10.1512213	42.254928	35.164383	1	2.323974599	3.886499986	0.166865696
74091	Camkv	235604	1.2072196	1	3.86389824	4.6248815	1	1	3.845915355	0.906117363
242608	Tpsb2	17229	4.19235242	6.11712518	22.8419672	16.4044191	1	1.203392024	3.806825893	0.213724896
16803	Ecm1	13601	3.0415586	3.60197733	12.7320939	12.5332494	1	2.375262236	3.802996424	0.508052078
319616	Dgka	13139	8.10697559	9.87251326	36.1289069	31.7604952	1.9042834	8.062672377	3.775936157	0.554351453
207683	Upb1	103149	1.49772727	1.21835768	5.13999623	5.08027925	1	1.633486294	3.762870324	0.969589073
218772	Gm7849	665927	1	1	3.31970191	4.1861375	1.980189583	1.117453455	3.752919703	1.548821519
15957	Itgb5	16419	4.041436	4.50884245	15.078526	16.9225843	2.976683225	5.778078483	3.742698032	1.023915392
16979	Gpcpd1	74182	12.9225824	13.2136671	51.5032297	45.5093318	3.542714484	6.324641913	3.711801167	0.377535284
71951	Igsf11	207683	2.23211197	2.26210297	8.54445693	8.07532431	2.161731885	2.816091479	3.698038805	1.107606875
332397	Col9a3	12841	1	1	2.53039446	4.84430332	1	1	3.687348887	1
14645	Arl4a	11861	4.97846435	5.14308501	19.4151911	17.7793293	3.962435084	3.365444814	3.674785261	0.723987962
21906	Asb4	65255	8.11911512	7.65085881	30.1224352	27.7603394	1	4.911351914	3.670441993	0.374848553
14159	Tle2	21886	1	1	3.28706389	4.04938692	1	1	3.668225408	1
68519	Mtss1l	244654	1.40529633	1.04110205	4.5467529	4.42113259	1	1	3.665750249	0.817528336
494504	Defa21	66298	25.7844971	20.3965466	88.8304873	79.7596625	6.609135619	3.774819864	3.650635336	0.224853201
12286	4933406C10Rik	74076	2.13412459	1.77408946	7.26203911	6.79075508	1	1	3.595707407	0.511742698
16909	Nanos1	332397	4.35559858	5.54743478	19.9332272	15.531734	2.077071817	1.913916209	3.581222031	0.403006622
330256	Fes	14159	1.21068847	1	3.43021214	4.41506323	1	3.308068939	3.548792824	1.948745381
17132	Nuak1	77976	1.24251079	1.26618885	4.6710999	4.17408708	1	1	3.525805494	0.797225768
83767	Ifit1	15957	1.21805085	1.12344766	3.76149872	4.49178695	1	1.400901674	3.524787929	1.025369723
16552	Lypd6b	71897	1.41581454	1.63291916	5.46039531	5.23389797	1	1	3.507782023	0.656010067
54396	Stx1a	20907	4.10102063	3.33330262	11.7316717	14.2773646	4.773572266	4.057832459	3.498507594	1.187923155
234912	Vash2	226841	2.54864194	2.48832857	8.83630735	8.66140699	4.371350319	3.316318339	3.473856816	1.526248495
22776	Adra2a	11551	2.76002072	1.83987704	7.57010127	8.38459965	1	1.050045963	3.468490335	0.445672071
22354	6430573F11Rik	319582	3.31208776	3.03409824	10.7993008	10.8505916	5.804186593	2.819161642	3.411480908	1.358823746
66684	Gsdma	57911	7.67043289	6.55436811	24.6065757	23.4424069	5.058127839	6.422036212	3.377831625	0.8070527
108899	Stxbp4	20913	1.36835551	1.37317456	4.76482425	4.4144044	1.937365425	2.004888076	3.348213748	1.43797566
226123	Pdgfrl	68797	3.37443345	2.91528555	10.7493551	10.1748738	1	1.578693892	3.326735093	0.409985548
14724	Gm20362	1.01E+08	1.5063007	1.24047757	3.9502586	5.08619403	1	2.251763015	3.289836944	1.183846199
72821	Gpc2	71951	2.44886832	2.03382747	6.6901706	8.04124508	1	2.250395074	3.286284942	0.725098295
83395	Ldhb	16832	1.83103466	2.04948524	6.74593445	5.94157	1	1.276877523	3.269537274	0.586745483
212190	Ccdc136	232664	2.36131367	2.48609909	8.11383182	7.71747926	1.249785667	2.248361007	3.265930064	0.721652322
11749	Ccdc141	545428	5.44972866	6.27460221	19.3144327	18.7816382	4.791568522	14.07689751	3.249317282	1.609342677
15507	Sidt1	320007	3.11659697	2.6939831	9.92765238	8.67627632	1	4.875029672	3.201733474	1.011091768
272636	Rps6ka2	20112	2.58259693	2.49749727	8.42374346	7.81850461	1.369449266	1.146741743	3.197233637	0.495304006

109624	Igtp	16145	1.60883341	1.47265528	5.2501513	4.58443582	1	1	3.191505181	0.649036943
68312	Fmnl3	22379	2.03191394	2.13260947	6.47072157	6.75380207	1.836454688	3.640139148	3.1755191	1.315058962
50781	Ifi27l1	52668	6.80259029	6.80048198	19.5864659	23.5238876	5.078491406	3.483118365	3.169163015	0.62938795
22361	Kif19a	286942	1.35128202	1	2.84433738	4.47867258	1	2.103762436	3.114475385	1.320029846
224344	Hr	15460	2.11592413	1.82053447	5.64566216	6.60622909	2.811704928	4.272836164	3.112414603	1.799724524
75986	Gdf11	14561	4.82932716	2.66074232	12.131625	11.0184723	1.399311795	2.425432786	3.090772029	0.51064207
15460	Onecut2	225631	3.23857869	3.65730235	11.2113282	10.0082755	1	5.923996548	3.077141782	1.004077145
22352	Wfdc15b	192201	3.89075463	3.27879318	11.2401571	10.7380439	1.326623907	2.44250345	3.065493336	0.525713401
18710	Gm1821	218963	5.5696227	4.24605424	15.6368716	14.2080238	14.18462396	5.256868695	3.040533584	1.980657348
171543	Abi2	329165	3.80963188	3.80135062	11.9881696	11.0277358	4.007726339	4.897182632	3.02403867	1.17000781
20907	Med13	327987	13.899821	14.463512	46.1876423	39.5133264	9.480343283	14.07298048	3.021540833	0.830414527
24088	Stx11	74732	2.60222047	2.58951856	7.25455439	8.3006597	2.252901909	5.288211214	2.996147145	1.452521606
319582	Tmem236	625286	1.5540433	1.82485807	5.20252142	4.88804172	1.831504251	1	2.986344392	0.837995533
71296	Defa2	1E+08	28.9056378	20.1168986	73.6978333	72.0584643	13.530357	8.636297849	2.97325084	0.452172745
22051	Entpd8	72090	7.95525734	6.66233085	19.9647707	23.2848874	14.51921652	6.662669819	2.958741043	1.449068483
14778	Irgm2	54396	1.88691986	1.52908156	5.32601208	4.72876976	1	1	2.943436085	0.585479851
117600	Srgap1	117600	2.06034609	2.14194094	6.40913874	5.84605904	2.229970925	1.966474802	2.916316207	0.99860997
76453	Ddx58	230073	5.79999919	5.45272728	17.2703809	15.4679028	5.238428224	6.25180942	2.909364573	1.021106989
17131	Gadd45g	23882	2.95549893	1.5990945	5.00201887	8.15732651	2.0602874	3.812898165	2.889246992	1.289508196
77579	Nrp2	18187	1.38877345	1.72908735	4.59742806	4.36322992	2.212455748	3.388218231	2.873976286	1.796319448
67194	Cdc42ep3	260409	2.80193871	3.12177033	8.73334117	8.2746902	3.037536195	4.197752187	2.871179401	1.221411844
382073	Mapk8ip1	19099	2.57617679	2.06066473	6.33722395	6.95553072	3.026674226	2.950144663	2.866769247	1.28898494
328949	Slc46a3	71706	1.19774188	1.66435471	4.32749454	3.81406968	1.591347711	1	2.844615462	0.905401906
11657	G630055G22Rik	414127	38.1493799	35.2726974	96.9147265	111.725601	44.78595298	55.84822795	2.841656568	1.370625631
29811	Lect2	16841	8.73150897	7.40352532	24.6066849	21.1061276	12.52500088	2.730532825	2.833140087	0.945491248
19224	Rps19-ps3	277692	1.99808374	1.74340511	5.04063433	5.55789662	2.466541395	1.933300583	2.832704141	1.175960201
381524	Pycr1	209027	24.2743036	25.7547209	73.3538	68.2890803	4.472115039	6.733222782	2.831214118	0.22397674
52250	Nfkbiz	80859	2.15374926	1.97389329	5.94550972	5.67351057	1.617476599	5.392392833	2.814928896	1.698274341
1E+08	Fgd2	26382	1.55697135	1	3.1162373	4.07561451	1.350795614	1.068371651	2.812644656	0.946106519
17161	Ceacam10	26366	4.72654372	4.00070014	12.3143622	12.177669	1	9.169746178	2.806387855	1.165287271
74182	Bmf	171543	3.14141991	2.85138115	8.45923558	8.28840672	1.290082564	3.008041769	2.794626775	0.717214586
414127	Vipr1	22354	5.34377254	4.84969494	14.6504391	13.6907701	5.985003953	4.273490751	2.78033057	1.006379304
102294	Rarb	218772	2.31575078	2.12230455	6.26164015	6.05159442	4.274359937	2.884260557	2.77446621	1.613008391
20349	Gstm7	68312	24.182123	21.3536161	68.3108103	57.9240672	5.778277137	10.65590337	2.772215405	0.360907297
56847	Lypd6	320343	6.18619796	5.84878193	18.1505455	15.2127461	1	1	2.772193382	0.166182247
74732	Zfp945	240041	1.60449018	1.3778168	4.15998867	4.04021061	1.542050487	1.206172359	2.749616102	0.921509043
67937	Extl1	56219	4.11010429	3.70427062	9.95984299	11.5006484	1.091742031	1.344873082	2.746283827	0.311811903
209027	Utp14b	195434	4.93713878	5.86021624	15.4243277	14.0592848	3.805791959	7.087333806	2.730632864	1.008869834
73747	Tmcc3	319880	5.40210357	5.27691228	14.6210498	14.5130195	8.83030335	11.85332004	2.728160507	1.936847335
107373	Tspyl2	52808	3.95415384	3.72358683	10.1452605	10.793142	2.202053316	5.118970882	2.72715677	0.953538875
14621	Ccdc112	240261	1.4623687	1.99498894	4.84809712	4.53286282	2.728276002	3.573091106	2.713332232	1.822596258
17752	Vnn1	22361	5.04685627	7.76602149	18.1780432	16.5550516	2.046643181	3.924585799	2.710795763	0.4660334
244853	Dnaja4	58233	4.41718054	4.26425141	11.7149655	11.6691454	1.473088595	2.247440622	2.693577626	0.428561698
20913	2700081O15Rik	108899	2.57019585	2.04383226	5.88915645	6.52805132	1.319279094	2.2145941	2.691185978	0.765897629
18164	Pnliprp2	18947	20.6146062	19.893486	61.7121477	47.1256953	1	11.81130444	2.686817302	0.316265312
319909	Adamts17	233332	5.00099305	4.60295009	13.513074	12.2772931	1.615007092	1.506710298	2.685393563	0.325045384
67216	Ptpre	19267	7.23905812	6.57482867	19.3976638	17.53297	4.775386157	7.107802526	2.673442626	0.860234984
12945	Bmp2	12156	1.70067504	1.8396473	5.10483348	4.35241282	1.179647679	4.425037557	2.671295261	1.583100265
228608	9230110C19Rik	234912	2.80698159	2.29016001	7.05642522	6.55763044	4.218929559	2.825406407	2.670919653	1.382016927
240261	Fam46a	212943	8.68964958	7.02962485	20.7288963	20.8989811	5.471644123	12.05440553	2.648206033	1.114940115
67855	Smad7	17131	2.94728791	2.80306539	7.36900432	7.83390971	1.165053011	2.486106672	2.643822602	0.634945279
12156	Ccdc84	382073	2.13341052	1.47455813	4.41775986	5.08846989	2.20812983	1.957756041	2.634787237	1.15463472
11982	Gulo	268756	8.10785057	8.21842535	23.1941553	19.7174899	1.728276564	2.088450428	2.628379271	0.233778175
52668	Ldlrad3	241576	3.08964975	2.92418077	8.28817233	7.43436541	2.453392911	2.597062799	2.614396545	0.839806791
18576	Ada	11486	6.45349818	6.73193012	17.7100367	16.5552857	12.86094724	10.55879431	2.598726536	1.776183604
380694	Fgfbp3	72514	1.69655048	1.37204004	3.62990955	4.3146502	2.06498056	1.635531108	2.588993122	1.205932054
382245	Ccnjl	380694	2.46881494	1.99487817	5.82846265	5.72215707	3.284740045	3.105993047	2.587682315	1.431714263
226841	Grik5	14809	1.84536955	1.66132496	4.07830084	4.98416904	1	1.51972295	2.584334002	0.718546465
239673	Fermt2	218952	4.79076252	5.20704184	14.0538237	11.7356797	1	4.590780011	2.579516711	0.559200781
74076	Cd200	17470	1.51926781	1.34423412	4.09371197	3.28548897	1	1	2.576984802	0.698445488
114606	Alb	11657	3.44764828	5.07201777	13.4203398	8.50590063	1	1.085338713	2.573603279	0.244767659
382083	Tlr2	24088	3.77144399	3.07683291	8.38418761	9.20335067	5.325717118	5.917364642	2.568169853	1.641738779
328957	Tgfb2	21808	3.80413066	4.01907333	10.9518091	9.06822068	1	1	2.55905762	0.255649732
241576	Sectm1a	209588	1.62726284	1.70600875	4.65058047	3.79057996	2.597365421	1.273593555	2.532395033	1.161309201
240041	Bex4	406217	10.5323729	7.14375102	21.342743	23.1273009	2.816348175	15.71909005	2.515825535	1.048614409
1E+08	Cetn2	26370	29.7842763	29.4682197	76.2954808	72.3724146	49.99123628	32.57691311	2.509057092	1.39349656
229927	Pcgf1	69837	5.66997864	5.58169696	14.83985	13.2055327	7.311400089	6.532134966	2.492551661	1.230353198
76263	Smox	228608	14.3422851	14.4400477	32.6685926	39.0305801	15.80904923	25.67788727	2.491082749	1.441402849
70355	Atxn7l2	72522	1.89403199	1.50119808	3.77596921	4.6741392	3.783920558	2.594988239	2.488817615	1.878785431
72022	Hist3h2a	319162	2.8764875	1.8053428	5.43500627	6.20899728	3.769151801	2.626434125	2.487062286	1.366043945
12578	Pmp22	18858	1.85502252	2.72287037	6.01791613	5.33192985	6.268947292	1.711920256	2.479272945	1.743349559
76260	Ptpro	19277	8.05546014	6.91268555	18.5570696	18.3425651	13.54351021	6.900737716	2.465210817	1.36585041
233332	Clk4	12750	8.22702598	8.69527467	20.9954092	20.6742917	7.787916414	10.16238203	2.46241346	1.060748111
20311	Nkd1	93960	57.5229596	50.7271147	129.351682	136.996289	11.84820416	18.33891651	2.460487644	0.278864665
19099	Gm20559	330256	10.8532333	9.93352457	26.7681031	24.2743877	6.553058905	11.78494973	2.455529188	0.882196672
56219	Slc35f2	72022	5.77655876	6.04902658	14.8256248	14.1795761	6.975955321	5.128336754	2.4527497	1.023568113
18947	Ttc8	76260	1.49132273	1.69941892	4.29814603	3.47644123	1	2.344436982	2.436608198	1.048169156
14174	Trim12c	319236	2.00289737	2.10045765	5.07861532	4.89209019	1.113966942	1.133318183	2.429891014	0.547670165
319162	Pglyrp1	21946	168.424826	161.208835	372.190133	428.367435	240.0037271	183.2980973	2.428628091	1.284158369
74050	LOC100862126	1.01E+08	9.79703911	7.65243189	21.2325038	20.7981457	3.416813255	2.848784912	2.408706224	0.359070952
230073	Bmp1	12153	2.9239941	2.38283904	6.1741092	6.55930063	1.636837383	2.743457449	2.399436633	0.82540655
71146	Rbm11	224344	3.9034135	5.00840212	10.6128115	10.6065775	2.648831918	1.024973871	2.381039951	0.412239878
72522	Gstk1	76263	3.21222511	3.51472594	8.68234891	7.3080444	2.618836515	5.725027011	2.377064021	1.240363348
80859	Cyp4v3	102294	2.23232364	2.24302547	5.31913628	5.30171934	1.613493348	2.353530256	2.373190421	0.886416571
23972	Plekhd1	217682	1.75196842	1.75539901	4.26567958	4.03334328	2.164375776	3.742571815	2.36616865	1.6841542
218963	Nrip1	268903	12.7759069	13.4130433	32.0622619	29.7290818	4.820183267	15.65963846	2.359443325	0.782002394
236733	Rfxank	19727	5.4504638	5.21084268	12.4404925	12.6264048	10.70228687	6.968343778	2.351203141	1.657454522
56213	Dmbt1	12945	3.88929887	6.57093194	14.1631592	10.4119544	1	1	2.34938541	0.19120037
18858	Ppp2r3d	19054	1.46823813	1.69418028	4.05607576	3.36015783	2.533408634	1.395534161	2.345114608	1.242385508

192216	Tcf12	21406	39.5945511	37.9044414	96.0417642	85.6622698	22.47248872	28.63427161	2.344598659	0.659450641
58194	Reep1	52250	2.38618202	2.31789417	5.43811138	5.58301333	1.203958505	2.368016117	2.342888219	0.759336046
286942	Slc23a2	54338	4.68811513	5.02772764	11.9638739	10.749063	2.954352762	4.423913877	2.337721739	0.759405726
20897	Nts	67405	6.11337048	6.6493407	15.0560686	14.7640661	5.063417692	3.006572817	2.336504705	0.632310047
20249	Myh10	77579	5.45882974	4.90790832	12.5843186	11.496994	10.7827346	7.578531905	2.322940206	1.771171066
217682	Rundc3a	51799	1.60720769	1.64383162	3.33857743	4.21187611	2.499958594	1.902919062	2.322473775	1.354298499
209773	Nox1	237038	11.5817319	8.55132809	27.9421998	18.6623143	4.839575138	1.846985128	2.314825176	0.33211843
71706	Pik3r3	18710	14.2135261	13.9760348	34.3114312	30.7264247	11.52427031	11.97676355	2.307161012	0.833678607
12583	LOC671650	671650	5.16598803	4.50562424	8.78542026	13.5196163	6.349313001	4.8079037	2.30623767	1.153604631
14026	Lrrc45	217366	21.5706688	21.6348918	47.3886514	52.0593719	12.18525046	15.38021856	2.301741304	0.638007439
16391	Gjb4	14621	3.1514411	2.74446644	7.25807266	6.23400538	4.804960929	4.574261045	2.288380192	1.590802078
16145	Pde3b	18576	5.7216161	5.20019347	12.6975953	12.2749199	2.447711967	5.555053092	2.286481469	0.732732521
13139	Ppap2b	67916	3.43414368	3.45408432	8.15275918	7.59374419	1	2.878457478	2.286002058	0.56305591
71897	Irf9	16391	3.76552468	3.91732221	8.5038279	9.03890744	1.389805928	3.424782099	2.283363913	0.626667184
75723	Smpd1	20597	2.46784588	2.63241199	5.50010629	6.14476399	6.019719235	3.942541387	2.283192452	1.95328567
58233	Usp11	236733	2.59638909	1.97689329	5.38329841	4.96943385	1.91259459	2.527502116	2.263742186	0.970877443
14268	Arl6	56297	4.61466622	4.35679853	10.4571536	9.79818003	9.734822677	4.828076716	2.257751012	1.623246572
1E+08	1110034G24Rik	73747	2.84450299	3.13507987	6.80543933	6.68734663	2.137663101	1.841572379	2.256476122	0.665470413
11861	Papss2	23972	10.2950384	12.390597	27.1822693	24.0061975	3.158817964	10.31401898	2.256426411	0.593892862
14465	Fn1	14268	6.01117658	6.30225575	15.749169	12.0040073	3.110805296	17.20735324	2.253894412	1.650080822
545428	D0H4S114	27528	2.16564039	1.98152793	4.71355545	4.62976254	1.793835305	1.143862602	2.252939181	0.708362353
1E+08	Arhgap42	71544	12.8626233	14.6067489	32.0867997	29.7617138	12.90394867	9.950450885	2.251544488	0.831995701
319236	Gata6	14465	6.85455543	6.41011216	14.9605883	14.8474256	8.760278284	7.777380377	2.247173831	1.24674505
21406	Msi1	17690	3.26005793	2.65730919	5.9693796	7.2336771	4.834523829	2.997666318	2.231238395	1.323593753
1E+08	Clk1	12747	19.5980607	18.4582132	43.0344463	41.441577	16.92678412	20.37882548	2.219766006	0.980274885
14561	Zswim5	74464	2.87434174	2.79305716	6.51252182	6.01937292	2.195044214	1.909465429	2.211225105	0.724231647
329165	Cyp27a1	104086	8.71450005	9.44227172	20.8093558	19.3214371	6.464290352	6.860425697	2.210238331	0.733870328
14618	Arl3	56350	11.9960389	11.5425142	25.0811397	26.9281796	26.81888401	12.61402681	2.209537652	1.675247867
75957	Cblb	208650	2.35995328	2.14891765	4.80939693	5.13589604	2.451087352	5.128333199	2.205716933	1.681001887
26366	Gprc5c	70355	3.31582668	2.34798534	6.458768	5.96347519	1	3.658966229	2.193265444	0.822584897
17229	Efcab4b	381812	4.49458496	3.99530073	8.92560338	9.62325861	1.073458792	3.47455881	2.184818815	0.535698332
225631	Gjb1	14618	3.6841002	3.9336012	7.75098575	8.8776375	1	4.460667769	2.1828925	0.716839304
13370	Mir17hg	75957	5.55692319	4.74098885	10.9659409	11.5121293	1.725405847	4.747973227	2.182779397	0.628610834
30946	Pea15a	18611	25.9186559	24.3941651	57.1515016	52.6167414	22.01687794	39.12338787	2.181715136	1.2152025
215707	Scd1	20249	82.4858644	86.4182613	189.476091	178.192462	86.94848591	185.3763	2.176788467	1.612303932
245386	Afm	280662	5.53466501	6.77539238	14.3906914	12.3752717	1	1	2.174316679	0.162468779
12841	8430419L09Rik	74525	2.77559909	2.3504549	5.59040189	5.54413274	1	1.876450197	2.172145405	0.561143172
20597	Fam89a	69627	2.99370673	1.97809395	5.70668013	5.09166111	5.593328255	1.991316526	2.171917566	1.525532752
72514	Amt	434437	19.1374977	16.6815958	40.3304193	37.3847894	12.36172868	23.77919548	2.169658724	1.008984891
103149	Il17rd	171463	2.6152584	2.74852862	5.99438688	5.6344848	1	1.92298872	2.168033824	0.544948692
14809	Hist2h2be	319190	4.64932249	4.1290589	9.64394418	9.32982616	5.453702488	3.4120479	2.161420138	1.009952745
320007	Znrf3	407821	11.6022073	10.9370109	25.0686257	23.5842448	5.733176921	9.819895691	2.158587308	0.6900449
14186	Amotl1	75723	6.92136314	7.11515951	15.5586578	14.6733838	2.018873058	10.55275144	2.153812757	0.895636677
68891	Npy	109648	40.0684084	44.911447	91.7129784	91.1373616	1	4.84348341	2.15169041	0.06876316
17427	H2-Q4	15015	8.8892426	7.4919704	17.2035262	17.963986	7.520681499	12.38435648	2.14681979	1.215113801
56350	2010111I01Rik	72061	3.32666041	3.10559651	6.98801158	6.81972895	5.956975395	4.385970778	2.146640086	1.607980877
11486	Zfp566	72556	2.33253819	2.67643868	5.54453972	5.18249996	2.812326899	2.161847115	2.14156303	0.993051904
21809	Ephb6	13848	1.60253432	1.89974674	3.48781568	4.00798331	1.384118679	1.281938899	2.140261982	0.761234618
77976	Muc6	353328	9.17059029	7.87518601	16.5808987	19.8646711	1	8.961394187	2.138099735	0.584390761
280662	Ctxn1	330695	8.83467461	7.04331985	15.9292176	18.0006812	15.6286082	10.3520989	2.136913379	1.636271329
12747	Tnfrsf11b	18383	17.2773925	19.4902582	39.5935165	38.6724162	37.30158425	20.71292508	2.128662862	1.577868266
195434	Stxbp1	20910	2.68305086	2.41759939	5.43998171	5.39452862	1.15954578	3.379219348	2.124142964	0.889840493
406217	Peli1	67245	4.19949049	4.30303329	9.30484746	8.74937219	5.34388292	3.971686637	2.123395375	1.095624053
69837	Sectm1b	58210	10.8414093	8.29159988	21.8731971	18.7428833	12.49304283	8.512518332	2.122827623	1.09787023
16419	Dtx3	80904	4.93903195	4.04193914	8.61938359	10.3603483	1.14246082	3.704013097	2.113327357	0.539638071
667373	Nrbp2	223649	3.17962296	3.25725688	6.5908713	6.98949247	1	2.218381361	2.109774319	0.499990903
17470	Mex3b	108797	2.89043614	2.3405948	5.3729075	5.63084875	2.882954764	2.38623826	2.103554035	1.007295327
68797	Sh3d21	66938	3.05432779	2.65755638	5.80857188	6.20426281	1.551917322	1.933242344	2.103129953	0.610159373
434437	Lypd8	70163	37.7641654	40.2840967	80.4631325	82.7166412	105.9523664	24.64354672	2.090754737	1.67327125
12750	Pdgfa	18590	13.4239561	8.95414512	21.2241901	25.3984051	13.57097999	11.74387001	2.083402641	1.131233152
72556	Hebp2	56016	4.10911811	3.0063936	7.35286909	7.46752416	3.66282278	9.674226395	2.082828875	1.87436262
268903	E2f2	242705	3.26963728	3.0870377	6.64072244	6.59627845	3.365687196	4.457069014	2.082378118	1.230636494
29867	Gpc1	14733	10.9333477	11.2068677	22.6700866	23.3920355	7.71447725	11.92026166	2.080473083	0.886835949
260409	Efemp2	58859	3.39156466	3.24101635	6.629309	7.16211856	1.406744575	3.455855991	2.079345509	0.73313851
27528	Atp6v0e2	76252	10.3381232	9.11443104	20.8561361	19.4871202	19.23234798	10.0061009	2.073931051	1.503064763
51799	Cpne2	234577	3.30496593	2.96808478	6.21262856	6.79562385	3.450791243	4.854999005	2.073672447	1.324043218
209588	Rnf24	51902	2.10649764	2.02226099	4.42591732	4.13000835	1.426426094	2.97710045	2.072275578	1.066549765
268756	Atp8b2	54667	2.2347174	1.94708194	4.27478363	4.35368793	1	2.94672106	2.063339453	0.943785376
241275	Csrnp2	207785	2.44462589	2.18586833	4.37712323	5.1352019	2.65650237	2.144515435	2.054278589	1.036826218
26370	Sh3bp2	24055	2.7038777	2.4339464	4.94770471	5.60434784	2.473157639	2.972183522	2.053797936	1.059853558
68511	Gramd3	107022	3.47146758	3.04450419	6.41952477	6.95150618	8.395475277	4.274754594	2.05203942	1.944488144
66793	Gm8979	668108	24.6539414	24.9969644	53.841459	47.9496507	4.496706742	2.148520437	2.050136008	0.133838992
19054	Ift80	68259	4.10141293	4.14271738	8.47141637	8.42524711	5.030036957	4.988722082	2.049538625	1.215259666
1E+08	Slc16a10	72472	9.61736184	9.48496862	20.8130357	18.3202261	4.192225728	2.703584597	2.048611913	0.360993144
21886	Sntb1	20649	8.61858006	7.8035993	17.9629326	15.6720717	1.808612701	7.091509242	2.048144989	0.541957419
18611	Myb	17863	13.8592069	11.8530826	25.767531	26.822288	22.23528051	17.86213468	2.045318401	1.559464984
19267	Arl5b	75869	7.77563501	7.6887347	15.5346949	16.0858136	4.408828016	9.35382281	2.044733089	0.88995873
67709	Plekhb1	27276	2.09274626	2.23492141	4.54789675	4.24129843	1	1.053307533	2.030931173	0.474460538
69453	Mapk4	225724	3.53389065	3.19536536	6.95105953	6.71139979	1	1	2.030307554	0.297209676
319880	Tmem56	99887	6.21460458	6.314047	13.2754513	12.1353398	7.102856802	3.560438419	2.028214362	0.851112759
71544	Lmo7	380928	36.6206375	41.9853468	81.4443097	77.9461413	44.34412271	71.70098335	2.027713952	1.476288442
20666	Sypl	19027	167.287983	164.71833	349.169806	323.940716	61.79436156	77.86215123	2.027402782	0.420644149
14677	Zfp866	330788	5.50841975	4.72950829	10.3172802	10.409703	2.854367512	4.343841914	2.024529095	0.703092403
12153	Ctns	83429	3.96501741	4.05595993	8.10586248	8.04312979	3.945024336	5.151860395	2.013344707	1.134136695
18187	Tet2	214133	3.41931255	3.09657653	6.94597876	6.15530779	1	3.613309162	2.010667526	0.708009162
27078	Sesn3	75747	10.2552233	8.90690743	19.3256897	19.1466727	11.31344187	9.541081449	2.007728839	1.088319646

[a]Gene gymbols of the selected 8 genes for expression analyses were highlited with yellow.
