# Supplementary material for: Intestinal cancer progression by mutant p53 through the acquisition of invasiveness associated with complex glandular formation
Source: Oncogene. 2017 Jun 19;36(42):5885–96. doi: 10.1038/onc.2017.194 (PMC5658682; doi:10.1038/onc.2017.194)
Supplement: Supplementary Table 2 [file onc2017194x9.pdf]

Supplementary Table 2. Clinicopathological information of human CRC samples

Case No.	*TP53* gene	Age	Gender	Tumor site（primary）	Materials	pStage	Genetic alteration (ex. driver gene)	Trial name
1	Wild	50	F	Rs	Primary tumor	IV	APC Q541fs, APC Q1294fs, FBXW7 S582L, ERBB2 Amplification	GI-SCREEN
2	Wild	66	M	Rb	Primary tumor	IIIB	KRAS G12V, APC E1309fs	GI-SCREEN
3	Wild	60	M	Rb	Lymph node	IIIC	KRAS G161L, SMAD4 R361S	GI-SCREEN
4	R273H	65	F	Sigmoid colon	Primary tumor	IIIC	-	GI-SCREEN
5	R273H	66	F	Rb	Metastatic tumor (Liver)	IV	APC K993fs, APC E1309fs, WT1 R434H	GI-SCREEN
6	R273C	43	M	Rectum (Ra、Rb)	Metastatic tumor (Liver)	IV	APC N933fs, TSC2 V339fs	BREAC
7	R273C	73	F	Sigmoid colon	Primary tumor	IV	APC K226fs, PIK3CA F83I	BREAC
8	P278S	70	M	Sigmoid colon	Primary tumor	IV	AR L55Q, BRCA2 S3041L, GNAS E481D, PIK3CA G460R	BREAC
9	V272M	64	F	Sigmoid colon	Primary tumor	IV	FLT3 P211T, TSC1 G965S, TSC1 G1016S	BREAC
